# The logic behind neural control of breathing pattern

**DOI:** 10.1038/s41598-019-45011-7

**Published:** 2019-06-24

**Authors:** Alona Ben-Tal, Yunjiao Wang, Maria C. A. Leite

**Affiliations:** 1 School of Natural and Computational Sciences, Massey University, Auckland, New Zealand; 2 Department of Mathematics, Texas Southern University, Houston, TX USA; 3 Mathematics and Statistics Unit, University of South Florida St Petersburg, St Petersburg, FL USA

**Keywords:** Applied mathematics, Dynamical systems

## Abstract

The respiratory rhythm generator is spectacular in its ability to support a wide range of activities and adapt to changing environmental conditions, yet its operating mechanisms remain elusive. We show how selective control of inspiration and expiration times can be achieved in a new representation of the neural system (called a Boolean network). The new framework enables us to predict the behavior of neural networks based on properties of neurons, not their values. Hence, it reveals the logic behind the neural mechanisms that control the breathing pattern. Our network mimics many features seen in the respiratory network such as the transition from a 3-phase to 2-phase to 1-phase rhythm, providing novel insights and new testable predictions.

## Introduction

The mechanism for generating and controlling the breathing pattern by the respiratory neural circuit has been debated for some time^1–6^. In 1991, an area of the brainstem, the pre Bötzinger Complex (preBötC), was found essential for breathing^7^. An isolated single PreBötC neuron could generate tonic spiking (a non-interrupted sequence of action potentials), bursting (a repeating pattern that consists of a sequence of action potentials followed by a time interval with no action potentials) or silence (no action potentials)^8^. These signals are transmitted, through other populations of neurons, to spinal motor neurons that activate the respiratory muscle^4,6^. The respiratory muscle contracts when it receives a sequence of action potentials from the motor neurons and relaxes when no action potentials arrive^9^. Hence, the occurrence of tonic spiking, bursting and silence can be associated with breath holding, breathing and no breathing (apnoea) respectively^10^. Tonic spiking, bursting and silence also appear in a population of coupled preBötC neurons when it is isolated *in vitro*^11,12^. Breathing can be performed with different combinations of frequency and amplitude to meet the body metabolic needs. However, the abilities to hold the breath and not to breathe are also important for supporting other activities such as diving, vocal communications and eating. Hence, we also expect to find tonic spiking, bursting and silence in a population of coupled preBötC neurons when it is embedded in the brainstem. The occurrence of bursting in the preBötC when this population interacts with other populations of neurons in the brainstem has been studied experimentally^6,13^. It was found that the preBötC population is activated during inspiration for about a third of the respiratory cycle, while two other distinct populations of neurons (called post-I and aug-E) are active consecutively during the remaining expiratory time of the cycle^13^. This was called a 3-phase pattern. A change in the conditions of the brainstem such as decreased carbon dioxide, transforms the 3-phase into a 2-phase pattern of inspiration and expiration where only one population of expiratory neurons (aug-E) remains active^6^. In extreme conditions of hypoxia (lack of oxygen), and despite being embedded in the brainstem where it could potentially interact with other populations of neurons, only the preBötC population remains active, generating a 1-phase pattern, similar to the pattern generated by the isolated preBötC population^6,14^. When the preBötC population activates the respiratory muscle, the 3-phase pattern leads to a normal breathing pattern while the 1-phase pattern leads to gasping - a breathing pattern with an abrupt inspiration. These findings illustrate the state-dependency and incredible plasticity of the respiratory neural network which are essential for survival. However, the existence of multiple mechanisms for generating breathing also makes understanding how the neural system works more difficult and may explain why it remains elusive.

Many of the experimental studies of the respiratory neural network were accompanied by theoretical studies using mathematical and computational models^8,11,15–19^. These models rely on differential equations with parameters that cannot always be measured directly and need to be estimated. Additionally, none of the existing models provide a clear understanding of how selective control of inspiration and expiration times can be achieved. In order to translate the models from the animal on which the experiments where based to humans, the models need to be re-scaled. This is because respiratory rates differ significantly across species. However, our impaired understanding of neural control of breathing and our inability to measure or estimate parameters in human models, make the translation from animals to humans difficult. The aim of this study is to unravel the logic behind the operation of the respiratory neural network and to provide a general mathematical framework for the study of neural control of breathing in all mammalian species. We do this by using Boolean networks in which the nodes could have only two values: “1” or “0”^20–24^. Our approach stems from the observation that the amplitude of action potentials is not functionally important - control signals stimulating the neural system (called tonic drive) convey information by changing the rate (frequency) of the action potentials, not their amplitude. We represent an action potential by “1” and the time that passes between action potentials by a sequence of “0”s. This allows us to generate signals that consist of spiking at various frequencies as well as other types of signals or patterns such as bursting, a critical characteristic of the respiratory rhythm generator. The control signals that stimulate the neural system, arrive from other brainstem regions and are regulated by chemoreceptors (which sense blood partial pressures of O_2_ and CO_2_) and mechanoreceptors (which sense lung inflation)^6,25^. Variations in the spiking frequency of control signals result in adjustments to the breathing pattern and ensure that blood gas partial pressures are maintained at the same levels. The Boolean network we present in this paper enables us to explore a key question for understanding control of breathing: how are the activation and quiescent times in a bursting signal changed *selectively* by varying the rate of tonic spikes in a control input signal? Such control of timing is crucial for supporting a wide range of activities involving breathing with diverse and dynamic combinations of inspiration and expiration times.

## Results

### Notation and framework setup

**Figure 1:**
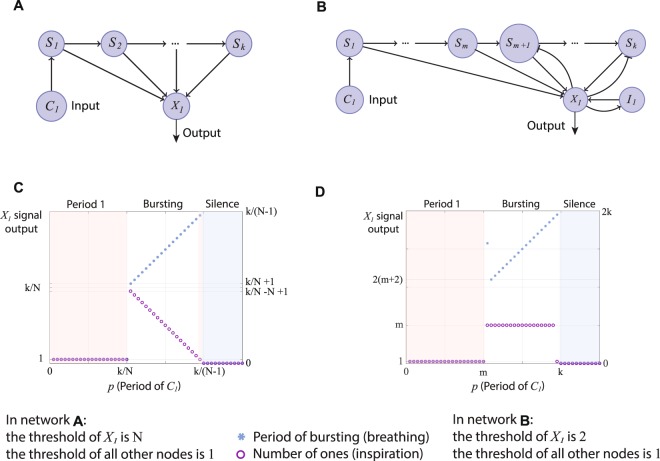
Examples of Boolean networks and their characteristic response to an input signal *C*_1_. An action potential (spike) is represented by “1” and the time that passes between action potentials is signified by a sequence of zeros. Panel (A) shows a network where the memory (represented by ${S}_{1},{S}_{2},\mathrm{\cdot \cdot \cdot },{S}_{k}$) is preserved after an action potential has been generated in ${X}_{1}$. Panel (B) shows a network with self excitation (depicted by ${I}_{1}$) where some of the memory is erased after a spike has been generated (the nodes ${S}_{1},{S}_{2},\mathrm{\cdot \cdot \cdot },{S}_{m}$ convey the memory that remains). Panel (C) shows the response of Network A and Panel (D) shows the response of Network B to changes in the period of ${C}_{1}$. In both networks when the period of ${C}_{1}$, $p$, is low (the spiking frequency is high), ${X}_{1}$ exhibits tonic spiking with period 1 (i.e. ${X}_{1}=111\mathrm{\cdot \cdot \cdot }$). When $p=k$/$N+1$ in Network A, $p=m+2$ in Network B, bursting appears (for example, ${X}_{1}=11100001110000\mathrm{\cdot \cdot \cdot }$). When $p\ge k$/$(N-1)$ in Network A, $p\ge k$ in Network B, ${X}_{1}$ exhibits silence (i.e. ${X}_{1}=000\mathrm{\cdot \cdot \cdot }$). The number of consecutive “1” within a burst is reduced in Network A as the period of ${C}_{1}$ increases but stays constant in Network B (the only exceptions are when $p=m+1$ and $p=k-1$ where we get different kinds of bursting, see Theorem 5). This characteristic response does not depend on the actual values of $k$ (memory size), $m$ (size of memory that was not erased) and $N$ (threshold of ${X}_{1}$ in Network A).

Figure 1, panels A and B, show two examples of Boolean networks that can produce bursting in response to a tonic spiking input. The node *C*_1_ denotes a control signal input and the node *X*_1_ signifies a neural output. The nodes *I*_1_ and ${S}_{1},{S}_{2},\mathrm{\cdot \cdot \cdot },{S}_{k}$ represent internal processes of the neuron *X*_1_. Connections between nodes could be excitatory (→) or inhibitory ($\dashv$). The states of all the nodes are updated simultaneously every step based on the current states of all the other nodes. This is done according to the following **Rules** (adapted with some modifications from Albert *et al*.^21,26^, see the Discussion for the neurophysiological interpretation):

- A node will be “1” in the next step if *N* activators are “1” in the current step and all the inhibitors are “0”; We call the number *N* a threshold and it can change from node to node;
- A node will be “0” in the next step if there are less than *N* activators which are “1” in the current step;
- A node will be “0” in the next step if at least one of the inhibitors is “1” in the current step, regardless of the state of the activators.

As the steps progress forward, we get a sequence of states for each node, called a trajectory. These trajectories could have certain characteristics:

(I) The trajectory $(000\mathrm{\cdot \cdot \cdot })\equiv (\bar{0})$ is silent.
(II) The trajectory $(111\mathrm{\cdot \cdot \cdot })\equiv (\bar{1})$ is tonic spiking with period 1.
(III) The trajectory $(10\cdots 010\cdots 0\mathrm{\cdot \cdot \cdot })\equiv (\overline{\mathop{\underbrace{10\cdots 0}}\limits_{p}})$ is tonic spiking with period *p*.

Where the over-line denotes a repeated pattern.

The trajectory of node *C*_1_ (the control input signal) is always tonic spiking with period *p*, where *p* is a control parameter. The trajectory of the output *X*_1_ could, in principle, be silent or tonic spiking and could also exhibit bursting or mixed mode oscillations. For example, the trajectories $(\overline{111000})$, $(\overline{10101000})$ or $(\overline{11101010010000})$ exhibit bursting and the trajectories $(\overline{1010100100})$ or $(\overline{11101010})$ exhibit mixed mode oscillations (a formal definition of bursting and mixed mode oscillations is given in the Methods).

There is one potential problem in the scheme we just presented here. If *C*_1_ = 0 in the current step, how do we know if it will be “1” or “0” in the next step? We show in Methods, Lemma 1, that this problem can be solved by keeping track of the states in another Boolean network, enabling us to know the value of *C*_1_ at every step. We further show in Lemma 1, that by increasing the number of connections in the network we can reduce the frequency of the spikes. This can be interpreted as changes in the internal properties of a neuron due to variations in a biophysical parameter.

### Characteristic response of minimal bursting networks

The Boolean networks we constructed in Fig. 1, panels A and B, represent some other properties of neurons. Specifically, the nodes ${S}_{1},{S}_{2},\mathrm{\cdot \cdot \cdot },{S}_{k}$ represent “memory” – the build-up of voltage potential in the neuron when it receives action potentials from external sources. The parameter *k* is the size of the memory. In network A (Fig. 1), the memory is preserved even after an action potential is generated in *X*_1_ (i.e. the state of *X*_1_ is “1”). However, an action potential will erase some of the memory in network B (Fig. 1) in the time step following *X*_1_ becoming “1”. The parameter *m* gives the size of the memory that remains. The node *I*_1_ represents self excitation – a property that has been shown to exist in certain neurons, including the preBötC^8^. We show in panels C and D (Fig. 1) that each of the networks A and B has its own characteristic response when the period of the input signal increases (the tonic spiking frequency *decreases*). In both networks, *X*_1_ exhibits spiking $(\bar{1})$ for a low period of *C*_1_ (high spiking frequency) and silence $(\bar{0})$ when the period of *C*_1_ is high (the spiking frequency is low). For mid-range values of *C*_1_, both networks exhibit bursting with increasing breathing (bursting) period as the period of *C*_1_ increases. However, in network A, an increase in the breathing period is always accompanied by a reduction in inspiration time (number of consecutive “1”s), while in network B, the inspiration time is constant. This characteristic response depends on neural properties such as the existence of memory, existence of a threshold and self excitation *regardless* of the actual values of these properties (provided the values are feasible). The actual values will affect the number of consecutive “1”s within a burst and the points where transitions from tonic spiking to bursting to silence occur but will not change the characteristic response qualitatively (see Methods for a proof of our general result).

### Constructing a larger bursting network

**Figure 2:**
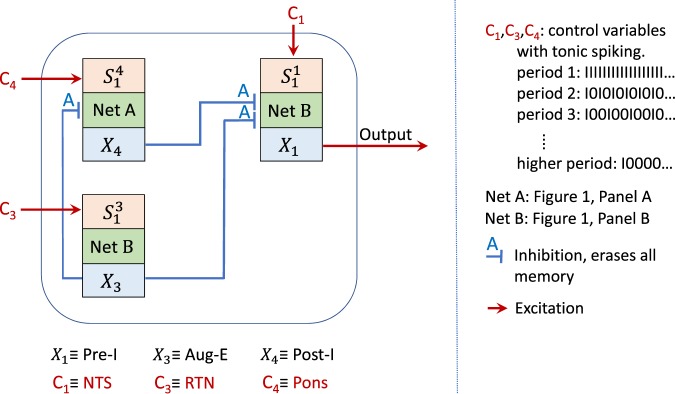
Schematic description of a larger network that provides better control of expiration and inspiration time. The Net A and Net B sub-networks are shown in Fig. 1, Panels (A and B) respectively. Here we only show the output and the first node to which the control input connects (${S}_{1}^{i}$, where $i$ is the sub-network number, this is equivalent to ${S}_{1}$ in Fig. 1, see Figs S9, S10 and S11 for more details). This structure can be related to the schematic representation of the respiratory neural network hypothesized in Smith *et al*.^6^. Sub-network ${X}_{2}$, is deliberately missing from our diagram. Unlike Smith *et al*.^6^, we found that this sub-network is not essential for generating and controlling the bursting signal.

While all the Boolean networks we found exhibit bursting (see the Supplementary Material and Methods), none allow for independent control of inspiration time and expiration time through variations in the period of *C*_1_. However, we can achieve independent control of the bursting pattern by connecting one type-A network (Fig. 1, Panel A) with two type-B networks (Fig. 1, Panel B). The structure of the larger network is shown in Fig. 2. The output of each sub-network is denoted by *X*_1_, *X*_3_ or *X*_4_ and the control input to each sub-network is denoted by *C*_1_, *C*_3_ or *C*_4_. For simplicity, we only show the first node to which the control input connects (${S}_{1}^{i}$, where *i* is the sub-network number, this is equivalent to *S*_1_ in Fig. 1). This structure can be related to a schematic representation of the respiratory neural network hypothesized by Smith *et al*.^6^. The central pattern generator in Smith *et al*.^6^ consists of four core populations of neurons: “pre-I” (a population of neurons within the PreBötC region, active during inspiration), “early-I” (a population of neurons within the PreBötC region, active during inspiration), “post-I” (a population of neurons within the BötC region, active in the first phase of expiration, under normal conditions) and “aug-E” (a population of neurons within the BötC region, active in the later phase of expiration, under normal conditions). The three populations, early-I, post-I and aug-E, mutually inhibit each other. Post-I and aug-E also inhibit pre-I, while pre-I excites early-I. In our large network, the sub-network *X*_1_ could represent pre-I, *X*_3_ could represent aug-E and *X*_4_ could represent post-I. Sub-network *X*_2_, which is missing from our diagram, could represent early-I. Unlike Smith *et al*.^6^, we found that this sub-network is not essential for generating and controlling the bursting signal. Another difference between our network and the network hypothesized in Smith *et al*.^6^ is that *X*_3_ is not affected by the other populations (see also the Section Mechanism of pattern generation). The control signals *C*_1_, *C*_3_ and *C*_4_ could represent, respectively, tonic drives from other brainstem regions such as the nucleus of the solitary tract (NTS; thought to be regulated by pulmonary mechanoreceptors and peripheral chemoreceptors^6^), RTN (thought to be regulated by central chemoreceptors, mainly sensitive to CO_2_^25^) and the Pons^6^. Note however, that our results do not depend on this interpretation.

### Transitions between states in the larger network

**Figure 3:**
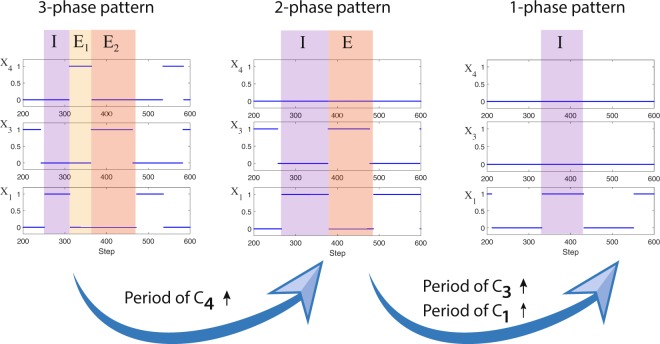
Transition from 3- to 2- to 1-phase pattern in the larger network (Fig. 2). In the 3-phase pattern, ${X}_{1}$ is active during phase “I” (inspiration), ${X}_{4}$ is active during phase “E_1_” (first phase of expiration) and ${X}_{3}$ is active during phase “E_2_” (second phase of expiration). In the 2-phase pattern, ${X}_{1}$ is active during inspiration, ${X}_{3}$ is active during expiration and ${X}_{4}$ is inactive. In the 1-phase pattern only ${X}_{1}$ is bursting. We used the following parameters to generate this figure. For ${X}_{1}$ and ${X}_{3}$, $k=400$, $N=2,$ $m=100$. For ${X}_{4}$, $k=800$, $N=3$. The 3-phase pattern is shown here when the period of ${C}_{1}=5$, the period of ${C}_{3}=110$ and the period of ${C}_{4}=32$. For the depicted 2-phase pattern, the period of ${C}_{4}$ is increased to $1000$. The 1-phase pattern is shown here when the period of ${C}_{4}=1000$ (same as for the 2-phase pattern), the period of ${C}_{3}=500$ and the period of ${C}_{1}=110$.

Figure 3 shows the output of the larger network. If the periods of ${C}_{1}$ and ${C}_{4}$ are low (tonic spiking frequency is high) and the period of ${C}_{3}$ is medium (relatively), the system exhibits a 3-phase pattern where ${X}_{1}$ is active during phase “I” (inspiration), ${X}_{4}$ is active during phase “E_1_” (first phase of expiration) and ${X}_{3}$ is active during phase “E_2_” (second phase of expiration). If the period of ${C}_{4}$ is large enough (tonic spiking frequency is low enough), ${X}_{4}$ becomes inactive and the system exhibits a 2-phase pattern where ${X}_{1}$ is active during inspiration and ${X}_{3}$ is active during expiration. If the period of ${C}_{3}$ is large enough (tonic spiking frequency is low enough) and the period of ${C}_{1}$ is medium (relatively), the system displays a 1-phase pattern where ${X}_{3}$ is inactive and only ${X}_{1}$ is bursting akin to gasping^14^. We refer to the periods of the control inputs as “low”, “medium” or “large” when, if acted on their respected *isolated* population, they result in tonic spiking, bursting or silence respectively (the actual values will depend on the size of the memory and threshold in each population). The transition from 3- to 2- to 1- phase pattern seen in our model is consistent with reducing energy due, for example, to lack of oxygen^6,14,27^. The system is also capable of displaying tonic spiking if the period of ${C}_{1}$ is low and the periods of ${C}_{3}$ and ${C}_{4}$ are high (not shown in the figure) and silence if the period of ${C}_{3}$ is low or if the periods of ${C}_{1}$, ${C}_{3}$ and ${C}_{4}$ are all high.

### Controlling inspiration and expiration times

**Figure 4:**
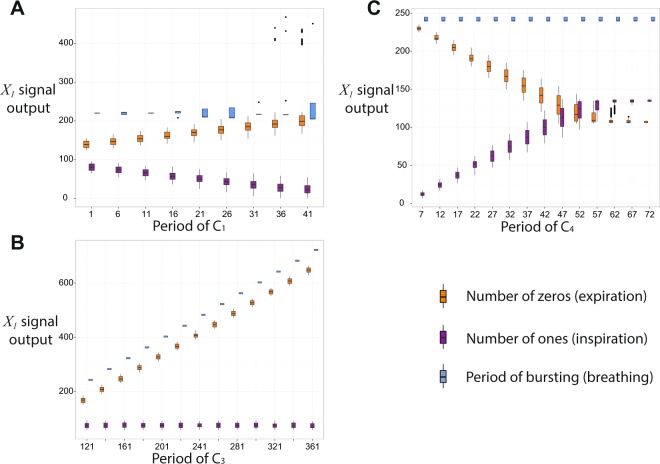
Controlling inspiration and expiration times within the 3-phase pattern. The period of breathing and expiration time can be increased (keeping inspiration time constant) by increasing the period of ${C}_{3}$ (RTN, Panel (B)). Expiration time can be decreased and inspiration time increase (keeping the period of breathing constant) by increasing the period of ${C}_{4}$ (Pons, Panel (C)). The inverse effect (increasing expiration time and decreasing inspiration time while keeping the period of breathing constant) can be achieved by increasing the period of ${C}_{1}$ (NTS, Panel (A)). This figure also shows that there is some variability in the timing of the bursting signals and that this variability increases when the periods of ${C}_{1}$ and ${C}_{4}$ increase. We used the following parameters to generate the figure. For ${X}_{1}$ and ${X}_{3}$, $k=400$, $N=2,$ $m=100$. For ${X}_{4}$, $k=800$, $N=3$. When it is not varied the period of ${C}_{1}=5$, the period of ${C}_{3}=110$ and the period of ${C}_{4}=32$.

The inspiration and expiration times within the 3-phase pattern can be controlled by varying the periods of ${C}_{1}$, ${C}_{3}$ and ${C}_{4}$ (see Fig. 4). The period of breathing and expiration time can be increased (keeping inspiration time constant) by increasing the period of ${C}_{3}$ (Panel B, Fig. 4, this result is consistent with experiments in which RTN was excited^28^). Expiration time can be decreased and inspiration time increased (keeping the period of breathing constant) by increasing the period of ${C}_{4}$ (Panel C, Fig. 4). The inverse effect (increasing expiration time and decreasing inspiration time while keeping the period of breathing constant) can be achieved by increasing the period of ${C}_{1}$ (Panel A, Fig. 4). Increasing the inspiration time while keeping expiration time constant can be achieved by tuning the periods of ${C}_{3}$ and ${C}_{4}$ simultaneously. Figure 4 also shows that there is some variability in the timing of the bursting signals. This is due to the order in which spikes arrive as inputs through the control signals ${C}_{1}$, ${C}_{3}$ and ${C}_{4}$ which can change depending on initial conditions and the periods of the control signals. This explains some of the inherent noise observed in the biological system. Our model predicts that the variability in inspiration and expiration times increases when the periods of ${C}_{1}$ and/or ${C}_{4}$ increase (tonic drive frequency from NTS and/or the Pons decrease).

**Figure 5:**
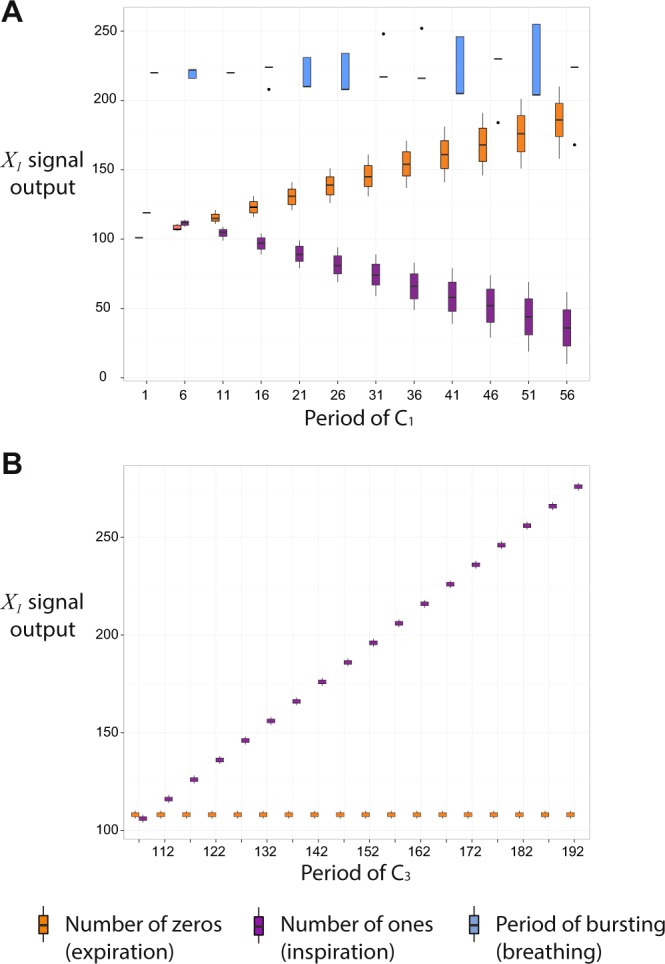
Controlling inspiration and expiration times within the 2-phase pattern. Increasing the period of ${C}_{1}$ (NTS, Panel (A)) results in increasing expiration time (number of “0”) and decreasing inspiration time (number of “1”s) while keeping the period of breathing constant on average. Increasing the period of ${C}_{3}$ (RTN, Panel (B)) increases the inspiration time (number of “1”s). The period of bursting is not shown in Panel (B) - its value increases as the period of ${C}_{3}$ increases with the same increasing tendency as the inspiration time. The parameters used to generate this figure are as follows: for ${X}_{1}$ and ${X}_{3},k=400,N=2,m=100$; for ${X}_{4},k=800,N=3$, and the period of ${C}_{4}=1000$. In panel (A) the period of ${C}_{3}=110$. In panel (B) the period of ${C}_{1}=5$.

When the system exhibits a 2-phase pattern, increasing the period of ${C}_{1}$ will have a similar effect to the one seen in Panel A, Fig. 4. That is, as the period of ${C}_{1}$ increases (tonic spiking frequency decreases), the expiration time increases, inspiration time decreases and the period of breathing stays constant (Panel A, Fig. 5). However, increasing ${C}_{3}$ *increases* the inspiration time while keeping the expiration time constant (Panel B, Fig. 5). This is the reverse effect of that seen in the 3-phase pattern and an interesting prediction of our model. This prediction can be tested by combining experiments where a 2-phase pattern was imposed by a ponto-medullary transection^29^, with experiments where RTN was excited^28^. Another prediction of our model is that the variability in the timing of the pattern gets larger as the period of ${C}_{1}$ increases but stays constant when ${C}_{3}$ is varied. When the system exhibits a 1-phase pattern (i.e. only ${X}_{1}$ remains active), it behaves like the isolated network in Panel D, Fig. 1, that is, as the period of ${C}_{1}$ increases (the frequency decreases) expiration time increases but inspiration time remains constant. This behavior is consistent with experiments described by Koizumi *et al*.^30^.

### Other types of patterns

**Figure 6:**
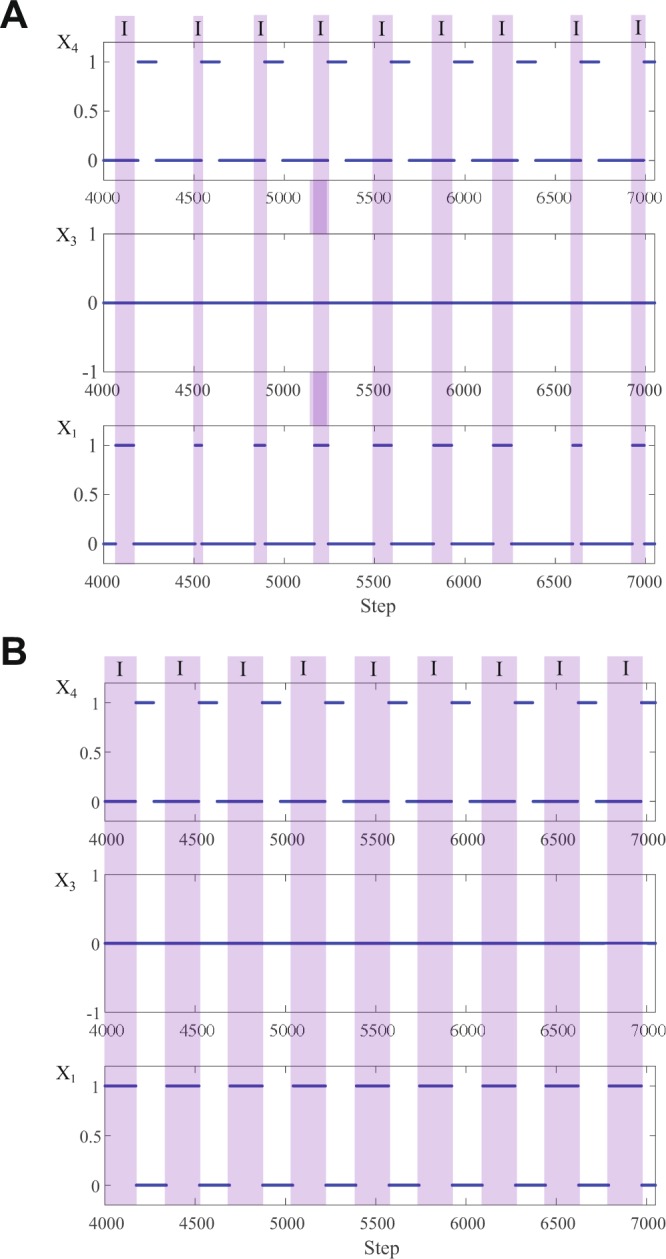
Other types of breathing patterns predicted by the model. Panel (A) shows periodic breathing - a dynamic change in inspiration time (marked by I) caused by an increase in the periods of ${C}_{1}$, ${C}_{3}$ and ${C}_{4}$ (decrease in the spiking frequency of NTS, RTN and the Pons respectively). As a result, ${X}_{3}$ (Aug-E) is silent and ${X}_{4}$ (Post-I) is bursting. ${X}_{1}$ (Pre-I) would have been bursting had it been in isolation. In Panel (B), the periodic breathing is suppressed by increasing the spiking frequency of NTS (decreasing the period of ${C}_{1}$). This leads to another way by which a 2-phase pattern can be achieved. We used the following parameters to generate this figure. For ${X}_{1}$ and ${X}_{3}$, $k=400$, $N=2$, $m=100$. For ${X}_{4}$, $k=800$, $N=3$. The period of ${C}_{3}=500$ and the period of ${C}_{4}=350$. The period of ${C}_{1}=110$ in Panel (A) and the period of ${C}_{1}=50$ in Panel (B).

Our model can produce other types of patterns. Figure 6, Panel A, shows dynamic changes in inspiration time. This situation can arise if the periods of ${C}_{1}$, ${C}_{3}$ and ${C}_{4}$ increase (the spiking frequency of NTS, RTN and the Pons *decrease* respectively) such that ${X}_{3}$ (aug-E) is silent and both ${X}_{4}$ (post-I) and ${X}_{1}$ (Pre-I) are in bursting states (when isolated). In Panel B, the periodic breathing is suppressed by increasing the spiking frequency of NTS (decreasing the period of ${C}_{1}$). This leads to another way by which a 2-phase pattern can be achieved where ${X}_{3}$ is silent and ${X}_{4}$ is bursting as opposed to the 2-phase pattern seen in Fig. 3 where ${X}_{3}$ is bursting and ${X}_{4}$ is silent. This new observation of our model is highly relevant for understanding the behavior of the respiratory system in disease states (for example, central sleep apnoea).

**Figure 7:**
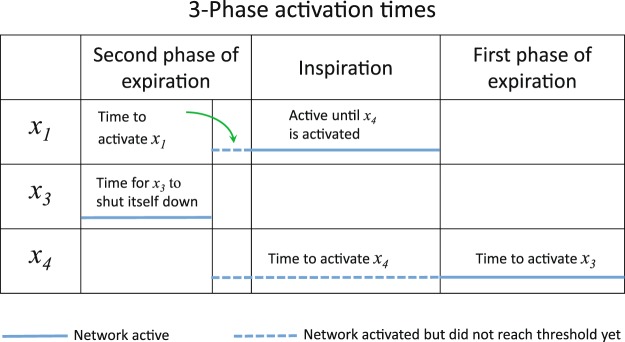
Mechanism of the 3-phase pattern generation. ${X}_{3}$ is in a bursting state due to the period of ${C}_{3}$. While it is active both ${X}_{1}$ and ${X}_{4}$ are inactive. When ${X}_{3}$ turns itself down (due to its bursting state), both ${X}_{1}$ and ${X}_{4}$ can be activated by their control signals ${C}_{1}$ and ${C}_{4}$ respectively. Due to the lower threshold of ${X}_{1}$ and the higher frequency of its control signal, ${X}_{1}$ is activated first. When ${X}_{4}$ is activated, it terminates ${X}_{1}$. When ${X}_{3}$ becomes active again (due to its bursting state), ${X}_{4}$ is terminated.

### Mechanism of pattern generation

In the larger network we presented in Fig. 2, bursting in the 3-phase pattern and in the 2-phase pattern is driven by the activity of ${X}_{3}$. The behavior of ${X}_{3}$ is determined *only* by the controller ${C}_{3}$; different periodic signals of ${C}_{3}$ lead to different patterns in ${X}_{3}$ as shown in Fig. 1, Panel D.

1. When ${X}_{3}$ is in a bursting state, then if it is active, both ${X}_{1}$ and ${X}_{4}$ are inhibited and if it is quiescent, both ${X}_{1}$ and ${X}_{4}$ could in principle be activated, depending on the period of their control signals (${C}_{1}$ and ${C}_{4}$ respectively). If the threshold of ${X}_{1}$ is lower than the threshold of ${X}_{4}$ and the frequency of ${C}_{1}$ is higher than the frequency of ${C}_{4}$ then ${X}_{1}$ will be activated first.
  - When ${X}_{4}$ is activated, it terminates ${X}_{1}$. When ${X}_{3}$ becomes active again (due to its bursting state), ${X}_{4}$ is terminated. This creates the 3-phase pattern (see Fig. 7).
  - If ${X}_{4}$ is not activated because the period of ${C}_{4}$ is too high (frequency is low) then ${X}_{1}$ will stay active until ${X}_{3}$ becomes active again and we get a 2-phase pattern.
2. When ${X}_{3}$ is in a spiking state (the period of ${C}_{3}$ is low, see Fig. 1, Panel D), both ${X}_{1}$ and ${X}_{4}$ are inhibited all the time.
3. When ${X}_{3}$ is in a silence state (the period of ${C}_{3}$ is high, see Fig. 1, Panel D), the behavior of the network is controlled by ${C}_{1}$ and ${C}_{4}$.
  - If the period of ${C}_{4}$ is high and therefore ${X}_{4}$ is in a silence mode, we get a 1-phase pattern if the period of ${C}_{1}$ is such that ${X}_{1}$ is in a bursting state. Otherwise we could either get silence if the period of ${C}_{1}$ is high (frequency is low) or spiking if the period of ${C}_{1}$ is low (frequency is high).
  - If the period of ${C}_{4}$ is low and therefore ${X}_{4}$ is in a spiking state, ${X}_{1}$ is inhibited all the time.
  - If however, the period of ${C}_{4}$ is such that ${X}_{4}$ is in a bursting state then more complex dynamics can arise where the inspiration period varies over time (see Fig. 6).

Figure 7 can explain the control of timing within a 3-phase pattern seen in Fig. 4. An increase in the period of ${C}_{3}$ will increase the first phase of expiration due to the characteristic behavior of the ${X}_{3}$ sub-network seen in Fig. 1, Panel D, where only the number of “0”s increases as ${C}_{3}$ increases. An increase in the period of ${C}_{1}$ will increase the time to activate ${X}_{1}$, hence inspiration time will decrease and the second phase of expiration will increase. An increase in the period of ${C}_{4}$ will result in a longer time to activate ${X}_{4}$, therefore inspiration time will increase and the first phase of expiration will decrease. While the period of bursting is constant when viewing the ${X}_{3}$ signal, it is varied when viewing the ${X}_{1}$ signal. This is due to the different times at which a “1” can arrive from the control signal ${C}_{1}$ and because the period is measured from the beginning or end of a burst.

Within a 2-phase pattern, inspiration will end when ${X}_{3}$ is activated. Hence, an increase in the period of ${C}_{3}$ will increase the inspiration time (not the expiration time as was the case in the 3-phase pattern). An increase in the period of ${C}_{1}$ will decrease the inspiration time, as has been the case for the 3-phase pattern. This behavior is shown in Fig. 5.

## Discussion

We have developed a new framework for studying neural networks based on Boolean representation in which the nodes could have only two values: “1” (signifying an action potential) or “0”. Our main motivation was to find a network architecture that mimics the respiratory neural network and enables selective control of inspiration and expiration times. Such selective control of timings is clearly present in humans. For example, in experiments designed to investigate how breathing affects heart rate and blood pressure^31–35^, subjects were asked to breathe in a paced frequency with a given inspiration and expiration times. While these experiments were not designed to study the respiratory neural network, they demonstrate what the network can do. Taken together, these experiments illustrate the diverse ability of the respiratory neural system to operate at different breathing frequencies with different ratios of inspiration to expiration times. Importantly, these experiments also justify the type of model we present in this paper. Unlike previous models, which were based on differential equations, our model can be easily scaled to represent breathing rates of different species. This can be done by choosing different time intervals between consecutive steps. The Boolean trajectory generated as an output of our model can then be transformed into an analog signal and coupled to models of other organs such as the lungs or the heart^36^. Hence, the Boolean framework we propose could be used on its own or as part of an integrated model, significantly enhancing our understanding of pattern generation and control of breathing rhythm. This could lead to strategic improvements in the treatment of cardio-respiratory diseases^37^ and to advances in our understanding of abnormal oscillations in the neural system.

The states in the Boolean networks we propose change according to a set of **Rules**. These **Rules** can be related to the operation of neurons. **Rules** (a) and (b) incorporate the property of a threshold for generating an action potential in neurons. **Rule** (c) incorporates a property of inhibition in which activation is blocked^38–40^. The property of summation of inhibition and excitation signals, in which these signals effectively cancel each other if they arrive within a certain window of time, is taken into account indirectly in our model. Such summation, within our Boolean framework, can be replaced by an equivalent excitation signal with a lower spiking frequency. This can explain some of the differences between the structure we suggest for the respiratory neural network and the structure previously suggested by others^6,18^, specifically, the lack of inhibition to ${X}_{3}$ (aug-E) in our model does not necessarily contradict the role of inhibition shown by other studies^40^. The same argument could explain why ${X}_{2}$ (early-I) was not essential in our model but was essential in other models^18^.

While certain aspects of our model could be shown to be equivalent to previous models, the output of our larger network differs significantly from the outputs of previous models. For example, in the study of Rubin *et al*.^18^ changes to the drives of pre-I, aug-E and post-I (the equivalent of ${C}_{1}$, ${C}_{3}$ and ${C}_{4}$ in our model) yield simultaneous changes in ${T}_{I}$, ${T}_{E}$ and $T$ (inspiration time, expiration time and breathing frequency, respectively), hence it is not clear how inspiration and expiration times can be controlled selectively. In contrast, our model shows several cases in which inspiration time, expiration time or the period of breathing can be kept almost constant while changing one of the control variables (Figs 4 and 5 and Panel D, Fig. 1). Of these, two cases (Panel B, Fig. 4 and Panel D, Fig. 1) agree with experimental observations. We are not aware of direct experimental observations for the other cases shown in Figs 4 and 5. However, we note that when inspiration time is longer, the amplitude of breathing is larger. With this in mind, indirect evidence for the scenario described in Panel C, Fig. 4, does exist (i.e. increase in breathing amplitude while breathing frequency remains constant)^41^.

Another difference between the output of our model and the output of the model proposed in the study of Rubin *et al*.^18^ is that the rate of spikes within a burst changes in the model proposed by Rubin *et al*.^18^ but stays mostly constant in our model (there is only one example in Fig. 1, Panel D, where the active phase of the bursting consists of period 1 spiking followed by period 2 spiking when p = m + 1, see Theorem 5, c). We did not try to model changes in network activity within a burst although our framework allows for such modeling. This is left for future investigations.

The structure of the network we present is not unique. It is the simplest representation we found that can provide selective control of inspiration and expiration. Previous studies have demonstrated that sub-network ${X}_{1}$ (Pre-I) has the property of self-excitation^8^. It has also been demonstrated that neurons in the brainstem have diverse bursting properties^5^. For this reason, we chose sub-network ${X}_{4}$ (post-I) to be different from sub-network ${X}_{1}$. We chose sub-network ${X}_{3}$ (aug-E) to be the same as sub-network ${X}_{1}$ because we wanted to be able to change the expiration time without changing the inspiration time. Replacing sub-network ${X}_{4}$ (post-I) by a network of similar type to ${X}_{1}$ (Pre-I) and ${X}_{3}$ (aug-E) generates the same results for the 3-phase and 2-phase states as long as ${X}_{4}$ has a tonic spiking state and a quiescent state with an activation time longer than ${X}_{1}$ (see Fig. 7). A different behaviour could exist in the 2-phase scenario shown in Fig. 6, Panel B (in which ${X}_{3}$ is silent and ${X}_{4}$ is active). If we were to plot a bifurcation diagram for this scenario (a task we leave for future investigation), we expect it to differ depending on the properties of ${X}_{4}$ (however, we note that when $N=3$ and $m$ is large enough, the networks described in Fig. 1, Panels A and B have similar properties, see Section 2.3 in the Supplementary Material).

In addition to the predictions and insights highlighted so far in the Discussion, our model shows a transition from 3- to 2- to 1- phase pattern that is consistent with energy reduction (Fig. 3). The model also predicts which control inputs can move the larger neural network into tonic spiking or silence (see Transitions between states in the larger network). An interesting prediction of our model is the increase in inspiration time while expiration time remains constant when the period of ${C}_{3}$ is increased (frequency of RTN is decreased, Panel B, Fig. 5) which is the reverse effect of that seen in the 3-phase pattern. We suggested an experiment to test this prediction (see Controlling inspiration and expiration times). Our model explains some of the inherent noise observed in the neural system (due to the order in which spikes arrive as inputs through the control signals) and predicts when the variability increases or remains unchanged (Figs 4 and 5, see also Controlling inspiration and expiration times). Furthermore, our model predicts conditions that lead to dynamic changes in inspiration time (Fig. 6, Panel A) and suggests a way to suppress it (Fig. 6, Panel B, see also Other types of patterns).

Our results illustrate the potential of using the Boolean framework for studying neural networks. Importantly, unlike traditional models, they provide general results that are determined by properties of neurons and not by the exact values of these properties. This uniquely enables us to capture the logic behind the multiple operational states of neural networks and the formation of distinct breathing patterns. Our network mimics many features seen in the respiratory network. It provides novel insights and new testable predictions. This does not exclude the development of more complicated Boolean models in the future if it is found that additional complexity is needed. We have assigned certain areas of the brainstem to each of the control signals ${C}_{1}$, ${C}_{3}$ and ${C}_{4}$. This particular physiological interpretation seems to be consistent with current experiments but could change when other evidence becomes available.

## Methods

Our study is based on logical arguments that we describe below and in the Supplementary Material. All of our results can be explained without computers but we provide graphs and numerical simulations for illustration. These were done using programs we wrote in Matlab and R (available at https://github.com/YunjiaoWang8/Logic-Behind-Breathing-Patterns).

We use the standard convention to graphically represent a Boolean network. The network consists of nodes that can have only two values, “1” or “0” (called the *state of the node*), and arrows that are either excitatory (denoted by →) or inhibitory (denoted by $\dashv$). Throughout this paper, nodes that are marked with ${X}_{i}$ (where *i* is a positive integer) represent the output of a neuron and nodes that are marked with ${I}_{j}^{i}$ and ${S}_{j}^{i}$ (where *i* and *j* are positive integers) represent internal processes associated with neuron ${X}_{i}$. For simplicity, we omit the superscript when it is clear which neurons the nodes ${I}_{j}$ and ${S}_{j}$ are associated with. We also distinguish between two types of external inputs: node E which is always “1” and always excitatory, and node ${C}_{i}$ (where *i* is a positive integer), which is a control input.

The state of the node ${X}_{i}$ in the current step is given by its value and we denote the state of ${X}_{i}$ at the next step as ${X}_{i}^{+}$. Similar notation is used for all the other nodes. As the steps progress forward, we get a sequence of states for each node (called a *trajectory*). These trajectories can have certain characteristics as described in the first section of the Results. In this paper we limit the trajectory of node ${C}_{i}$ to being periodic tonic spiking. Lemma 1 shows how periodic tonic spiking can be produced by a Boolean network, effectively showing how to transform a nonautonomous network to an autonomous network.

Following a proof of Lemma 1 we give a formal definition of bursting and mixed mode oscillations and explain what we mean by steady state. We then provide more information about the dynamics of the networks shown in Fig. 1. Other types of networks and their dynamics are described in the Supplementary Material. In every network we introduce in this section the control signal ${C}_{1}$ is an input to the network and the output is the state of the node ${X}_{1}$. In all of the networks, the governing functions follow the **Rules** introduced in the first section of the Results. We assume that the symbols $k,m,p,r$ and $N$ are all positive integers.

### Tonic spiking network

#### Lemma 1

*For any given periodic signal* ${C}_{1}=(\overline{10\cdots 0})$ *with* $p-1$ *consecutive* “*0*”*s following a state of* “*1*”, *there exists a Boolean network that can generate the signal*.

**Figure 8:**
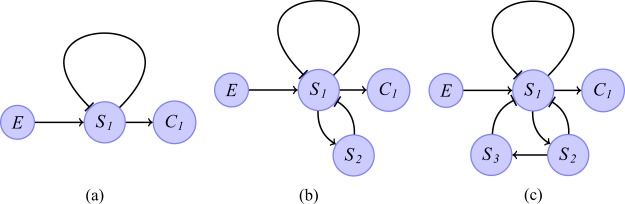
Creating a periodic signal: (**a**) ${C}_{1}=(\overline{10})$ (period 2); (**b**) ${C}_{1}=(\overline{100})$ (period 3); (**c**) ${C}_{1}=(\overline{1000})$ (period 4).

*Proof*. We prove the lemma by constructing networks that generate the signals. First we show that the networks in Fig. 8(a,b) generate the signals $(\overline{10})$ and $(\overline{100})$ respectively. In all the networks we construct in this proof, we assume that the threshold *N* to activate each node is one. By the **Rules**, the truth table for the network in Fig. 8(a) is shown in Table 1.

**Table 1:** Truth table of the network in Fig. 8(a), where * means the value is either 0 or 1.

$({S}_{1},{C}_{1})$	$({S}_{1}^{+},{C}_{1}^{+})$
$(0,\ast )$	$(1,0)$
$(1,\ast )$	$(0,1)$

This implies that ${C}_{1}=(\overline{10})$ (period 2). We look next at the truth table for the network in Fig. 8(b), Table 2.

**Table 2:** Truth table of the network in Fig. 8(b), where * means the value is either 0 or 1.

$({S}_{1},{S}_{2},{C}_{1})$	$({S}_{1}^{+},{S}_{2}^{+},{C}_{1}^{+})$
$(0,0,\ast )$	$(1,0,0)$
$(1,0,\ast )$	$(0,1,1)$
$(0,1,\ast )$	$(0,0,0)$
$(1,1,\ast )$	$(0,1,1)$

As can be seen we get the trajectory $({S}_{1},{S}_{2})=(1,0)\to (0,1)\to (0,0)\to (1,0)$ which gives ${C}_{1}=(\overline{100})$ (period 3).

We now assume that a network which generates a signal of period *p* exists and that it has the nodes ${S}_{1},\mathrm{\cdot \cdot \cdot },{S}_{p-1}$. We can always add another node, ${S}_{p}$ that is excited by ${S}_{p-1}$ and inhibits ${S}_{1}$, thereby creating a signal with period $p+1$ (see Fig. 8(c) as an example).$\square$

### Bursting, mixed mode oscillations and steady state

#### Definition 2

*Let a trajectory consist of a repeated pattern of period* $m+n$ *where the first* $m$ *steps of the pattern are composed of consecutive* “*1*”*s and* “*0*”*s that start and end with* “*1*”, *followed by* $n$ *consecutive* “*0*”*s*. *Let max*${(0)}_{m}$ *be the maximum number of consecutive* “*0*”*s within the first* $m$ *steps of the pattern*. *Then*, *a trajectory exhibits bursting if*:

1. *the trajectory is not periodic with period p*, *i*.*e*. *the pattern is not* $(\overline{\mathop{\underbrace{10\cdots 0}}\limits_{p}\mathop{\underbrace{10\cdots 0}}\limits_{p}})$;
2. $n > \,{\max }\,{(0)}_{m}$, *in other words*, *there are more consecutive zeros in the last* $n$ *steps of the pattern than there are in the first* $m$ *steps*.

*If* $n\le \,{\max }\,{(0)}_{m}$ *and the trajectory is not periodic with a period p then we say that the trajectory has mixed mode oscillations*.

We note that a trajectory of an autonomous and deterministic Boolean network with a finite number of nodes will eventually repeat a certain pattern. This is because a network with a finite number of nodes has a finite number of states, hence, the trajectory will eventually come back to one of the states it has visited before. By Lemma 1, our network models are equivalent to deterministic and autonomous systems. Hence, all trajectories will eventually repeat some pattern. We say that a trajectory is in *steady state* if it consists of a repeated pattern.

### Excitatory network

Consider the network shown in Fig. 1, panel A, where the node *X*_1_ is activated by the nodes ${S}_{1},{S}_{2},\mathrm{\cdot \cdot \cdot },{S}_{k}$. The integer *k* is a parameter that can be considered an internal property of the network, representing a memory length. The threshold for excitation of the nodes ${S}_{i}$ (where $i\in \{1,\mathrm{\cdot \cdot \cdot },k\}$) is one but the threshold, *N*, of ${X}_{1}$ could change (this is another internal property of the network representing how excitable a neuron is; low values of *N* indicate higher excitability). Theorem 3 characterizes the dynamics of networks with such topology.

Let $K=\{1,\mathrm{\cdot \cdot \cdot },k\}$, then the governing functions of the nodes in Fig. 1(A) when $N=2$ are:

1 $$\begin{array}{rcl}{S}_{1}^{+} & = & {C}_{1}\\ {S}_{i+1}^{+} & = & {S}_{i}\\ {X}_{1}^{+} & = & \mathop{\vee }\limits_{i,j\in K,i\ne j}({S}_{i}\wedge {S}_{j})\end{array}$$

#### Theorem 3

*Let the network in* Fig. 1, *Panel A*, *be governed by the* **Rules** *with* ${C}_{1}=(\overline{10\cdots 0})$ *being a periodic signal of period* $p$. *Then*

1. *when* $p\le \frac{k}{N}$, ${X}_{1}=(\bar{1})$ *at steady state*;
2. *when* $\frac{k}{N} < p < \frac{k}{N-1}$, ${X}_{1}=(\overline{\mathop{\underbrace{1\cdots 1}}\limits_{s}\mathop{\underbrace{0\cdots 0}}\limits_{p-s}})$ *at steady state*, *where* $s=k-(N-1)p$;
3. *when* $p\ge \frac{k}{N-1}$, *X*_1_ *is silent at steady state*.

#### **Remark**.

In the remaining of this paper, suppose ${C}_{i}$ has a period of *p*, then we can always assume that at steady state, if ${S}_{i}=1$, then ${S}_{i+1}=0,{S}_{i+2}=0,\mathrm{\cdot \cdot \cdot },{S}_{i+p-1}=0$ and ${S}_{i+p}=1$. i.e. “1” only shows up once every *p* nodes. This is because after the initial transient response which depends on initial conditions, the nodes $\{{S}_{i}\}$ will follow the input signal ${C}_{1}$ which is periodic with period *p*.

*Proof*.

1. When $p\le \frac{k}{N}$, i.e. $Np\le k$, there are at least *N* number of ${S}_{i}$ having state value “1” at any time step after the initial *k* time steps. Hence, ${X}_{1}$ has at lease N activators being “1” at any time step greater than *k*. By the **Rules** (a), ${X}_{1}=(\bar{1})$ at steady state.
2. When $\frac{k}{N} < p < \frac{k}{N-1}$, i.e. $(N-1)p < k < Np$, without loss of generality, we can assume that at Step 0,
  2 $$({S}_{1}\cdots {S}_{k})=(\mathop{\overbrace{\mathop{\underbrace{\overline{10\cdots 0}}}\limits_{p}}}\limits^{N-1}1\mathop{\underbrace{0\cdots 0}}\limits_{s-1})$$
  where $\mathop{\overbrace{\overline{\mathop{\underbrace{10\cdots 0}}\limits_{p}}}}\limits^{N-1}$ means $\mathop{\underbrace{10\cdots 0}}\limits_{p}$ repeats $(N-1)$ times and $s=k-(N-1)p$. Note that in this case, there are *N* number of “1”s in the values of $({S}_{1}\cdots {S}_{k})$. The number of “1”s will remain *N* until *s* time steps have passed, at this point
  3 $$({S}_{1}\cdots {S}_{k})=(\mathop{\underbrace{0\cdots 0}}\limits_{s}\mathop{\overbrace{\mathop{\underbrace{\overline{10\cdots 0}}}\limits_{p}}}\limits^{N-1})$$
  Note that now there are $N-1$ number of “1”s in the sequence $({S}_{1}\cdots {S}_{k})$. The number of “1”s will remain $N-1$ until $p-s$ time steps have passed, after this step the state values of $({S}_{1}\cdots {S}_{k})$ will change back to the initial state shown in Eq. (2). This pattern of changing from Eqs (2) to (3) and back to Eq. (2) repeats every *p* time steps. Over one period (i.e. *p* time steps), there are *s* time steps at which *N* number of ${S}_{i}$ have state value “1” and at the remaining $p-s$ steps, only $N-1$ number of ${S}_{i}$ have state value “1”. Hence, ${X}_{1}=(\overline{\mathop{\underbrace{1\cdots 1}}\limits_{s}\mathop{\underbrace{0\cdots 0}}\limits_{p-s}})$ at steady-state.
3. When $p\ge \frac{k}{N-1}$, i.e. $(N-1)p\ge k$, at any time step, there are at most $N-1$ number of ${S}_{i}$ having state value “1”. Hence, ${X}_{1}$ will never get activated. That is, ${X}_{1}$ is silent at steady state.$\square$

Theorem 3 is illustrated by the diagram in Fig. 1, Panel C. It shows that in the excitatory network periodic trajectories of period one persist for a range of control signals ${C}_{1}$. Furthermore, it shows a sudden transition in the trajectory of ${X}_{1}$ from periodic to bursting when $p$ (the period of the control signal ${C}_{1}$) changes from $p=\frac{k}{N}$ to $p=\frac{k}{N}+1$. As the period of ${C}_{1}$ increases further, the number of consecutive ones within one burst decreases until we get a periodic trajectory with period $\frac{k}{N-1}-1$ when $p=\frac{k}{N-1}-1$. For values of $p$ greater than $p=\frac{k}{N-1}-1$, silence persists. This network provides qualitatively the three types of signals seen in the PreBötC (where tonic spiking, bursting and quiescent signals exist). However, the range over which bursting is seen is highly dependent on $k$ which is a limiting feature. Furthermore, if the threshold $N$ is one, the network cannot be silenced and if $N$ is greater than 2 the range over which bursting is seen is reduced to $k/N < p < k/(N-1)$.

### Excitatory network with memory loss and self-excitation

Let the Boolean system associated with the network in Fig. 1, panel B, with $N=2$ be:

4 $$\begin{array}{cccc}{S}_{1}^{+} & = & {C}_{1} &\\ {S}_{i}^{+} & = & {S}_{i-1} & {\rm{f}}{\rm{o}}{\rm{r}}\,1 < i\le m\\ {S}_{i}^{+} & = & {S}_{i-1}\wedge ({\rm{\neg }}{X}_{1}) & {\rm{f}}{\rm{o}}{\rm{r}}\,m < i\le k\\ {X}_{1}^{+} & = & \mathop{\displaystyle \vee }\limits_{i,j\in K,i\ne j}(({S}_{i}\wedge {S}_{j})\vee ({S}_{i}\wedge {I}_{1})) &\\ {I}_{1}^{+} & = & {X}_{1} &\end{array}$$

where $$K=\{1,\mathrm{\cdot \cdot \cdot },k\}$$.

#### Remark 4

*In Theorem 5*, *the results about steady states of System (4) are presented for the initial condition*

$$({S}_{1}\cdots {S}_{k})=(1\mathop{\underbrace{0\cdots 0}}\limits_{p-1}10\cdots 0),\,{X}_{1}=0\,and\,{I}_{1}=0.$$

*which we call the* **feasible state**. *However*, *the results in Theorem 5 hold for other initial states for most combinations of the constants* $m$ *and* $k$ *as shown in Lemmas 6*–*8*. *Therefore*, *the results in Theorem 5 are rather general even though we fix the initial condition*.

#### Theorem 5

*For system (4)*, *suppose the signal* ${C}_{1}=(\overline{10\cdots 0})$ *is periodic with period p and* $m\ge 3$ *and suppose the initial state is the feasible state*

$$({S}_{1}\cdots {S}_{k})=(1\mathop{\underbrace{0\cdots 0}}\limits_{p-1}10\cdots 0),\,{X}_{1}=0\,and\,{I}_{1}=0.$$

*Then*

- when $p\le m$, ${X}_{1}=(\bar{1})$ at steady state;
- *when* $p=m+1$ *and* $p$ *is even*, ${X}_{1}=(\overline{10})$ *at steady state*;
- *when* $p=m+1$ *and* $p$ *is odd*, ${X}_{1}=(\overline{\mathop{\underbrace{1\cdots 1}}\limits_{m}\mathop{\underbrace{0101\cdots 01}}\limits_{m+1}\mathop{\underbrace{0\cdots 0}}\limits_{m+2}})$ *at steady state*;
- *when* $m+2\le p < k-1$, ${X}_{1}=(\overline{\mathop{\underbrace{1\cdots 1}}\limits_{m}\mathop{\underbrace{0\cdots 0}}\limits_{2p-m}})$ *at steady state*;
- *when* $m+2\le p=k-1$, ${X}_{1}=(\overline{\mathop{\underbrace{1010\cdots 10\cdots 0}}\limits_{2p}})$ *at steady state*, *where alternation between 1 and 0 occurs for a consecutive* $m$ *steps if* $m$ *is even or* $m+1$ *if* $m$ *is odd for every* $2p$ *steps*.
- *when* $p\ge k$, *then* ${X}_{1}$ *is silent at steady state*.
  *Proof*.
  - When $p\le m$, by the **Rules**,
    $$({S}_{1}\cdots {S}_{k})=(01\mathop{\underbrace{0\cdots 0}}\limits_{p-1}10\cdots 0),\,{X}_{1}=1\,{\rm{and}}\,{I}_{1}=0$$
    at Step 1 and at Step 2,
    $$({S}_{1}\cdots {S}_{k})=(001\mathop{\underbrace{0\cdots 0}}\limits_{p-1}\cdots ),\,{X}_{1}=1\,{\rm{and}}\,{I}_{1}=1.$$
    Since $p\le m$, there is at least one of ${S}_{i}$ with a value of “1” at any step after this. It follows that $({X}_{1},{I}_{1})=(1,1)$ for all the next steps as well. Hence, ${X}_{1}=(\bar{1})$.
  - When $p=m+1$ and $p$ is even, by the **Rules**,
    $$({S}_{1}\cdots {S}_{k})=(01\mathop{\underbrace{0\cdots 0}}\limits_{p-1}10\cdots 0),\,{X}_{1}=1\,{\rm{and}}\,{I}_{1}=0,$$
    at Step 1 and at Step 2,
    $$({S}_{1}\cdots {S}_{k})=(001\mathop{\underbrace{0\cdots 0}}\limits_{p-1}\cdots ),\,{X}_{1}=1\,{\rm{and}}\,{I}_{1}=1.$$
    The number of ${S}_{i}$ with a value of “1” remains one and the values of ${X}_{1}$ and ${I}_{1}$ also remain “1” until Step $p-1=m$ where
    $$({S}_{1}\cdots {S}_{k})=(\mathop{\underbrace{0\cdots 0}}\limits_{p-1}0\cdots ),\,{X}_{1}=1\,{\rm{and}}\,{I}_{1}=1.$$
    It follows that at Step $p=m+1$,
    $$({S}_{1}\cdots {S}_{k})=(1\mathop{\underbrace{0\cdots 0}}\limits_{p-1}0\cdots ),\,{X}_{1}=0\,{\rm{and}}\,{I}_{1}=1$$
    and at Step $p+1=m+2$
    $$({S}_{1}\cdots {S}_{k})=(01\mathop{\underbrace{0\cdots 0}}\limits_{p-1}0\cdots ),\,{X}_{1}=1\,{\rm{and}}\,{I}_{1}=0.$$
    The values of ${X}_{1}$ and ${I}_{1}$ alternate between “0” and “1” at least up to Step $2p-1$. Because $p$ is even,
    $$({S}_{1}\cdots {S}_{k})=(\mathop{\underbrace{0\cdots 0}}\limits_{p-1}10\cdots ),\,{X}_{1}=1\,{\rm{and}}\,{I}_{1}=0$$
    at Step $2p-1$. Then at Step 2*p*,
    $$({S}_{1}\cdots {S}_{k})=(1\mathop{\underbrace{0\cdots 0}}\limits_{p-1}00\cdots ),\,{X}_{1}=0\,{\rm{and}}\,{I}_{1}=1,$$
    which is the same value as the state value at Step $p$. Since ${C}_{1}$ is periodic of a period $p$, the pattern of the activity states from Step $p$ to $2p$ repeat every $p$ steps. Hence, ${X}_{1}=(\overline{10})$ at steady state.
  - When $p=m+1$ and $p$ is odd, by the **Rules**,
    $$({S}_{1}\cdots {S}_{k})=(01\mathop{\underbrace{0\cdots 0}}\limits_{p-1}10\cdots 0),\,{X}_{1}=1\,{\rm{and}}\,{I}_{1}=0$$
    at Step 1 and at Step 2,
    $$({S}_{1}\cdots {S}_{k})=(001\mathop{\underbrace{0\cdots 0}}\limits_{p-1}\cdots ),\,{X}_{1}=1\,{\rm{and}}\,{I}_{1}=1.$$
    The number of ${S}_{i}$ with a value of “1” remains one and the values of ${X}_{1}$ and ${I}_{1}$ also remain ‘1’ until Step $p-1=m$ where
    $$({S}_{1}\cdots {S}_{k})=(\mathop{\underbrace{0\cdots 0}}\limits_{p-1}0\cdots ),\,{X}_{1}=1\,{\rm{and}}\,{I}_{1}=1.$$
    It follows that at Step $p=m+1$
    $$({S}_{1}\cdots {S}_{k})=(1\mathop{\underbrace{0\cdots 0}}\limits_{p-1}0\cdots ),\,{X}_{1}=0\,{\rm{and}}\,{I}_{1}=1$$
    and at Step $p+1=m+2$,
    $$({S}_{1}\cdots {S}_{k})=(01\mathop{\underbrace{0\cdots 0}}\limits_{p-1}0\cdots ),\,{X}_{1}=1\,{\rm{and}}\,{I}_{1}=0.$$
    The values of ${X}_{1}$ and ${I}_{1}$ alternate between “0” and “1” for all steps between $p+1$ and $2p-2$. Because $p$ is odd, the number of steps between Step $p+1$ (inclusive) and Step $2p-2$, is an odd number ($p-2$ steps).
    At Step $$2p-2$$,
    $$({S}_{1}\cdots {S}_{k})=(\mathop{\underbrace{0\cdots 0}}\limits_{p-2}10\cdots ),\,{X}_{1}=1\,{\rm{and}}\,{I}_{1}=0.$$
    It follows that at Step $2p-1$,
    $$({S}_{1}\cdots {S}_{k})=(\mathop{\underbrace{0\cdots 0}}\limits_{p-1}00\cdots ),\,{X}_{1}=0\,{\rm{and}}\,{I}_{1}=1.$$
    Then at Step 2*p*,
    $$({S}_{1}\cdots {S}_{k})=(1\mathop{\underbrace{0\cdots 0}}\limits_{p-1}00\cdots ),\,{X}_{1}=0\,{\rm{and}}\,{I}_{1}=0.$$
    The number of ${S}_{i}$ with a value “1” remains one and the values of ${X}_{1}$ and ${I}_{1}$ remain “0” until Step $3p$, at which
    $$({S}_{1}\cdots {S}_{k})=(1\mathop{\underbrace{0\cdots 0}}\limits_{p-1}10\cdots ),\,{X}_{1}=0\,{\rm{and}}\,{I}_{1}=0,$$
    which is the same as the feasible state. Since ${C}_{1}$ is periodic with period $p$, the states from Step 0 to $3p$ repeat every $3p=3m+3$ steps. Hence, ${X}_{1}=(\overline{\mathop{\underbrace{1\cdots 1}}\limits_{m}\mathop{\underbrace{0101\cdots 01}}\limits_{m+1}\mathop{\underbrace{0\cdots 0}}\limits_{m+2}})$ at steady state.
  - When $m+2\le p < k-1$, by the **Rules**,
    $$({S}_{1}\cdots {S}_{k})=(01\mathop{\underbrace{0\cdots 0}}\limits_{p-1}10\cdots 0),\,{X}_{1}=1\,{\rm{and}}\,{I}_{1}=0$$
    at Step 1,
    $$({S}_{1}\cdots {S}_{k})=(001\mathop{\underbrace{0\cdots 0}}\limits_{p-1}\cdots ),\,{X}_{1}=1\,{\rm{and}}\,{I}_{1}=1$$
    at Step 2. The number of ${S}_{i}$ with a value of “1” remains one and the values of ${X}_{1}$ and ${I}_{1}$ remain “1” until Step $m\le p-2$ at which
    $$({S}_{1}\cdots {S}_{k})=(0\cdots 0),\,{X}_{1}=1\,{\rm{and}}\,{I}_{1}=1.$$
    It follows that at Step $m+1\le p-1$,
    $$({S}_{1}\cdots {S}_{k})=(0\cdots 0),\,{X}_{1}=0\,{\rm{and}}\,{I}_{1}=1.$$
    At Step $m+2\le p$, ${I}_{1}=0$, ${X}_{1}=0$ and the number of ${S}_{i}$ with a value of “1” is at most one. The values of ${X}_{1}$ and ${I}_{1}$ remain “0” up to Step $2p$ at which
    $$({S}_{1}\cdots {S}_{k})=(1\mathop{\underbrace{0\cdots 0}}\limits_{p-1}10\cdots 0),\,{X}_{1}=0\,{\rm{and}}\,{I}_{1}=0.$$
    That is, the state value is the same as the initial value. Since the period of ${C}_{1}$ is $p$, the pattern of states repeat every $2p$ steps. Hence at steady state, ${X}_{1}=(\overline{\mathop{\underbrace{1\cdots 1}}\limits_{m}\mathop{\underbrace{0\cdots 0}}\limits_{2p-m}})$.
  - When $m+2\le p=k-1$, by the **Rules**, at Step 1,
    $$({S}_{1}\cdots {S}_{k})=(01\mathop{\underbrace{0\cdots 0}}\limits_{p-1}),\,{X}_{1}=1\,{\rm{and}}\,{I}_{1}=0.$$
    At Step 2,
    $$({S}_{1}\cdots {S}_{k})=(001\mathop{\underbrace{0\cdots 0}}\limits_{p-2}),\,{X}_{1}=0\,{\rm{and}}\,{I}_{1}=1.$$
    Up to Step $m$ if $m$ is even, and up to Step $m+1$ if $m$ is odd, the number of “1”s in the sequence $({S}_{1}\cdots {S}_{k})$ remains one and the values of ${X}_{1}$ and ${I}_{1}$ alternate between “1” and “0”. From Step $m+2$ (≤*p*) to Step $k-1$ all ${S}_{i}$ have a value of “0” and the values of ${X}_{1}$ and ${I}_{1}$ are “0”. The values of ${X}_{1}$ and ${I}_{1}$ remain “0” until Step $2p$, at which the state goes back to the initial state. The pattern of activity repeats every $2p$ steps. Hence, ${X}_{1}=(\overline{\mathop{\underbrace{1010\cdots 010\cdots 0}}\limits_{2p}})$ at steady state, where alternation between 1 and 0 occurs for $m$ steps if $m$ is even or $m+1$ steps  if $m$ is odd for every $2p$ steps.
  - When $p\ge k$, with the feasible initial condition, the number of ${S}_{i}$ with a value “1” is at most one. That is, ${X}_{1}$ has at most one activator at any time step. Hence, by the **Rules** ${X}_{1}=(\bar{0})$.$\square$

#### Lemma 6

*For system (4)*, *suppose the signal* ${C}_{1}=(\overline{10\cdots 0})$ *is periodic of period* $p$ *with* $m+1 < p\le k-1$, *then all the trajectories pass through the state*

$$({S}_{1}\cdots {S}_{k})=(1\mathop{\underbrace{0\cdots 0}}\limits_{p-1}10\cdots 0),\,{X}_{1}=0\,{\rm{and}}\,{I}_{1}=0$$

*Proof*. When $m+1 < p\le k-1$, we first note that ${X}_{1}$ cannot be always zero at steady state. We can see this by contradiction. Suppose ${X}_{1}=0$ at steady state, then at least two ${S}_{i}$ have a value of “1” every $2p$ steps since $p\le k-1$. This means ${X}_{1}$ will be activated every $2p$ time steps, which is a contradiction.

We also note that ${X}_{1}$ cannot be always “1” at steady state. Again, we show this by contradiction. Suppose ${X}_{1}=1$ at steady state, then ${S}_{i}=0$ for all $i\ge m+1$. Since $p\ge m+2$, there exist at least two steps in every consecutive $p$ steps at which all ${S}_{i}=0$. It follows that ${X}_{1}=0$ every $p$ steps, which is a contradiction.

Next we show that a steady state cannot be $(\overline{10})$. Suppose ${X}_{1}=(\overline{10})$. Then at any step, ${S}_{i}=0$ for all $i\ge m+2$. Since $p > m+1$, there is at most one ${S}_{i}$ having value “1” for $i\le m$ at any given step. Moreover, $({S}_{1}\cdots {S}_{k})$ cannot be all zeros for two consecutive steps, otherwise, ${X}_{1}$ will be zero for two consecutive steps which is a contradiction.

Without loss of generality, we assume that at Step 0 the values of the signals are

$$({S}_{1}\cdots {S}_{m+2})=(\mathop{\underbrace{0\cdots 01}}\limits_{m}00)$$

Observe that the case ${X}_{1}={I}_{1}=1$ is not admissible. Otherwise $({S}_{1}\cdots {S}_{k})$ would all have values equal zero in the next step,

leading to ${X}_{1}$ being “0” for two consecutive steps, which is a contradiction. Hence the values of ${I}_{1}$ and ${X}_{1}$ can only be: (case 1) ${X}_{1}=1$ and ${I}_{1}=0$, and (case 2) ${X}_{1}=0$ and ${I}_{1}=1$.

Next we discuss case by case.

#### **Case 1**.

At Step 1,

$$({S}_{1}\cdots {S}_{m+2})=(\mathop{\underbrace{0\cdots 00}}\limits_{m}00),\,{X}_{1}=0\,{\rm{and}}\,{I}_{1}=1$$

At Step 2,

$$({S}_{1}\cdots {S}_{m+2})=(\mathop{\underbrace{0\cdots 00}}\limits_{m}00),\,{X}_{1}=0\,{\rm{and}}\,{I}_{1}=0$$

There are two consecutive “0”s in the trajectory of ${X}_{1}$, which is a contradiction.

#### **Case 2**.

At Step 1,

$$({S}_{1}\cdots {S}_{m+2})=(\mathop{\underbrace{0\cdots 00}}\limits_{m}10),\,{X}_{1}=1\,{\rm{and}}\,{I}_{1}=0$$

At Step 2,

$$({S}_{1}\cdots {S}_{m+2})=(\mathop{\underbrace{0\cdots 00}}\limits_{m}00),\,{X}_{1}=0\,{\rm{and}}\,{I}_{1}=1$$

At Step 3,

$$({S}_{1}\cdots {S}_{m+2})=(\mathop{\underbrace{0\cdots 00}}\limits_{m}00),\,{X}_{1}=0\,{\rm{and}}\,{I}_{1}=0$$

There are two consecutive “0”s in the trajectory of ${X}_{1}$, which is a contradiction. Hence, the trajectory of ${X}_{1}$ at steady state contains consecutive zeros. More specifically, the trajectory of ${X}_{1}$ contains the sequence 100.

Without loss of generality, suppose that ${X}_{1}=1,0,0$ at Steps 0, 1 and 2 respectively. Because ${X}_{1}$ inhibits all ${S}_{i}$ for $i > m$, there exists at most one ${S}_{i}$ with $i\le m$ having a value of “1” and ${S}_{i}=0$ for $i > m$ at Step 1. Then at Step 2, there is at most one of ${S}_{i}$ having value “1” while ${I}_{1}=0$. Because the threshold for activating ${X}_{1}$ is 2, ${X}_{1}$ will remain “0” up to the step at which

$$({S}_{1}\cdots {S}_{k})=(1\mathop{\underbrace{0\cdots 0}}\limits_{p-1}10\cdots 0),\,{X}_{1}=0\,{\rm{and}}\,{I}_{1}=0$$

The lemma is then proved.$\square$

#### Lemma 7

*When* $p < m$, *the steady state* ${X}_{1}=(\bar{1})$ *found in Theorem 5 is the only steady state of System (4) for any initial condition*.

*Proof*. We prove the lemma first by showing the result for the case $p\le m-2$ and then for the case $p=m-1$.

When $p\le m-2$, i.e., $p+2\le m$, $({S}_{1}\cdots {S}_{p+2})$ is determined only by the periodic control signal ${C}_{1}$.

For any given trajectory at steady state, there must exist a step at which

$$({S}_{1}\cdots {S}_{p+2})=(\mathop{\underbrace{10\cdots 0}}\limits_{p}10)$$

where there are at least two “1”s in the sequence $({S}_{1}\cdots {S}_{k})$. For convenience, we assume this occurs at Step 0. Because the threshold of ${X}_{1}$ is 2, ${X}_{1}=1$ at Step 1 and

$$({S}_{1}\cdots {S}_{p+2})=(0\mathop{\underbrace{10\cdots 0}}\limits_{p}1)$$

At Step 2, ${X}_{1}=1$ and ${I}_{1}=1$. Since $p+2\le m$, at any following steps, there is at least one ${S}_{i}$ with $1\le i\le m$ having a value of “1”, and ${X}_{1}$ and ${I}_{1}$ remain “1”. Hence, ${X}_{1}=(\bar{1})$ at steady state.

Next, consider $p=m-1$. All trajectories at steady state must pass through the state characterized by

$$({S}_{1}\cdots {S}_{p+1})=(\mathop{\underbrace{10\cdots 0}}\limits_{p}1)$$

and the value of ${X}_{1}$ can be either “1” or “0”. Without loss of generality, we assume it occurs at Step 0.

Suppose ${X}_{1}=1$. Then at Step 1,

$$({S}_{1}\cdots {S}_{p+1})=(0\mathop{\underbrace{10\cdots 0}}\limits_{p}0)$$

and $({X}_{1},{I}_{1})=(1,1)$. Since $p=m-1$, at steady state, there is at least one ${S}_{i}$ having a value of “1”, and both ${X}_{1}$ and ${I}_{1}$ remain “1” for all the steps afterward. Hence, ${X}_{1}=(\bar{1})$ at steady state.

Suppose ${X}_{1}=0$, then at Step 1,

$$({S}_{1}\cdots {S}_{p+2})=(0\mathop{\underbrace{10\cdots 0}}\limits_{p}1)$$

and ${X}_{1}=1$. At Step 2, $({X}_{1},{I}_{1})=(1,1)$. Again there is at least one ${S}_{i}$ having a value of “1”, and both ${X}_{1}$ and ${I}_{1}$ remain “1” for all the steps afterward. Hence, ${X}_{1}=(\bar{1})$ at steady state.$\square$

#### Lemma 8

*For System (4)*, *when* $p > k+1$, ${X}_{1}=(\bar{0})$ *for any initial condition*.

*Proof*. When $p > k+1$ there are at least two consecutive steps every $p$ steps at which all ${S}_{i}$ nodes have a value of “0”. This will result in ${X}_{1}$ and ${I}_{1}$ being “0” at the same step. Moreover, there is at most one of ${S}_{i}$ having a value of “1” at any step at steady state because of $p > k+1$. Hence, without a loss of generality, we can assume that at Step 0,

$$({S}_{1}\cdots {S}_{k})=(10\cdots 0)\,{\rm{and}}\,{X}_{1}=0,{I}_{1}=0$$

By the **Rules**, ${X}_{1}$ has at most one activator having a value of “1” at any step that follows. Hence, ${X}_{1}$ cannot be reactivated and ${X}_{1}=(\bar{0})$.$\square$

The results of Theorem 5 are illustrated in Fig. 1, panel D.

## Supplementary information

Supplementary Materials

## Data Availability

All data is available in the main text or the Supplementary Material. The codes used to perform the numerical simulations are available at https://github.com/YunjiaoWang8/Logic-Behind-Breathing-Patterns.
