# Supplementary material for: The logic behind neural control of breathing pattern
Source: Sci Rep. 2019 Jun 24;9:9078. doi: 10.1038/s41598-019-45011-7 (PMC6591426; doi:10.1038/s41598-019-45011-7)
Supplement: Supplementary file 1 — Supplementary Materials [file 41598_2019_45011_MOESM1_ESM.pdf]

Supplementary Materials for:
The logic behind neural control of breathing pattern

Alona Ben-Tal,[1*⋆] Yunjiao Wang,[2⋆] Maria C.A. Leite[3]

[1]School of Natural and Computational Sciences, Massey University,
Auckland, New Zealand, E-mail: a.ben-tal@massey.ac.nz
[2] Department of Mathematics, Texas Southern University,
Houston, TX, USA, E-mail: Yunjiao.Wang@tsu.edu
[3]Mathematics & Statistics Program, University of South Florida St. Petersburg,
St Petersburg, FL, USA, E-mail: mcleite@mail.usf.edu
[*]Correspondence author [⋆] Equal contribution.

Contents

1 Example illustrating the application of the Rules **2**

2 Other minimal bursting networks **2**

2.1 Inhibitory network 2

2.2 Excitatory network with memory loss 4

2.3 Excitatory network with memory loss and self excitation ($N > 2$) 17

3 Structure of the larger network **25**

1 Example illustrating the application of the Rules

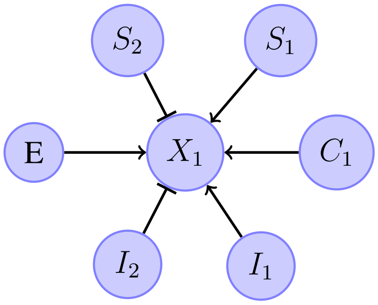

Figure S1: Typical inputs to node X_1. Node E is an external input - it is always "1" and always excitatory. Nodes I_i and S_i (where i is an integer) represent internal processes associated with neuron X_1 - they could be either "1" or "0", excitatory or inhibitory (in this example, nodes I_2 and S_2 are inhibitory). Node C_1 is a control input.

In the network shown in Fig. S1, if $N = 3, S_1 = 1, S_2 = 0, I_1 = 0, I_2 = 0$ and $C_1 = 1$, then $X_1^+ = 1$ since there are three excitatory signals with "1" in the current step. If in this same example, $C_1 = 0$, then $X_1^+ = 0$ since there are only two excitatory signals in the current step. On the other hand, if $N = 3, S_1 = 1, S_2 = 1, I_1 = 1, I_2 = 0$ and $C_1 = 1$, then $X_1^+ = 0$ because there is now one inhibitor with "1".

2 Other minimal bursting networks

2.1 Inhibitory network

Consider the network in Fig. S2 where the node X_1 is continuously excited by an external input and is inhibited by nodes S_1 to S_k. The integer k is a parameter that can be considered an internal property of the network, representing a memory length. The threshold for activation in all the nodes is one. Lemma 2.1 shows that X_1 can be either silent or exhibit bursting depending on the period of the control signal and the parameter k.

Lemma 2.1. *Let the network in Fig. S2 be governed by the* **Rules** *with* $C_1 = (\overline{10\cdots0})$ *being a periodic signal of period* p*. Then*

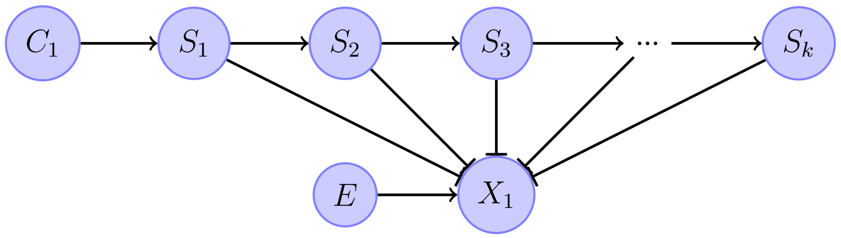

Figure S2: Inhibitory network.

1. *when $p \leq k$, X_1 is silent at steady state;*

2. *when $p > k$, $X_1 = (\overline{1 \cdots 10 \cdots 0})$ with $p - k$ consecutive "1"s and k consecutive "0"s at steady state.*

Proof. 1. Note that when $p \leq k$, after the first p steps, there is always at least one nonzero S_i (where $i \in \{1, \cdots, k\}$). Since an inhibitory signal dominates, node X_1 will be zero in the remaining time steps.

2. Without loss of generality we assume that the initial state (Step 0) is $(S_1, \cdots, S_k, X_1) = (0, \cdots, 0, 0)$ and $C_1 = 1$. Then, when $p > k$, there is exactly one nonzero S_i from Step 1 to Step k, and all the nodes S_i have a zero value from Step $k+1$ to Step p. By the **Rules** inhibitory signals dominate, it follows that $X_1 = 0$ from Step 2 to Step $k+1$ and will be activated again from Step $k+2$ to Step $p+1$. This means the trajectory is $(\overline{1 \cdots 10 \cdots 0}\ )$ with $p - k$ consecutive "1"s and k consecutive "0"s at steady state.

□

Lemma 2.1 describes a transition in the inhibitory network whereby, as the period of C_1, p, increases, X_1 moves from a silent state to a bursting trajectory via a single appearance of a periodic trajectory with period $k+1$ (which appears when $p = k+1$). When $p = k+2$, X_1 exhibits bursting with two consecutive "1"s. As p increases further, the number of consecutive

"1"s within a burst increases, but the number of consecutive "0"s stays the same (equal to k). This behavior is summarized schematically in Fig. S3.

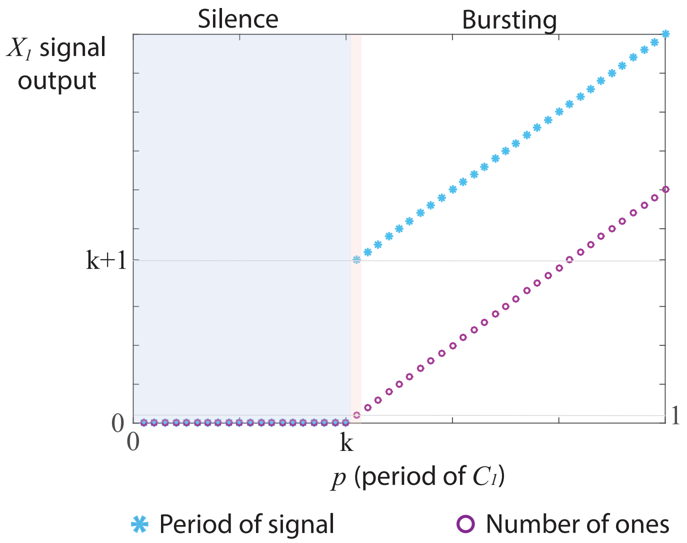

Figure S3: The output of the inhibitory network (X_1, Fig. S2) as a function of p. X_1 is silent when $p \leq k$, periodic with period $k+1$ when $p = k+1$ and bursting with period p and $p-k$ consecutive "1"s when $p > k+1$.

Although a periodic trajectory appears in the inhibitory network, it appears only when $p = k+1$. The networks we introduce next can exhibit all three states: silent, bursting and periodic trajectories over a range of p values.

2.2 Excitatory network with memory loss

Assume that once node X_1 is activated, the memory chain is partially erased. This mimics the idea that once a threshold is met and an action potential is generated, it takes time before the threshold can be met again (in other words, the buildup of current is removed when the action potential is generated). We realize this by the following set-up: besides the excitation from S_i to node X_1 as in Fig. 1, Panel A (main article), node X_1 inhibits the activity of some nodes, say $S_{m+1}, \cdots, S_k$ as indicated in Fig. S4. Note that when $m = k$, the network is the same as

the network in Fig. 1, Panel A (main article). That is, the network scheme in this section is a generalization of the one represented in Fig. 1, Panel A (main article). We start by considering first the threshold $N = 2$ for the excitation of X_1 and we also focus first on the case $m \geq 7$.

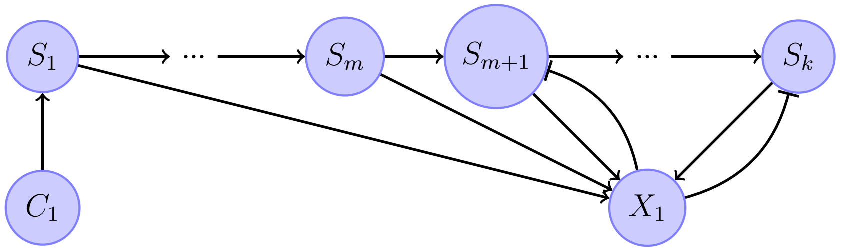

Figure S4: Excitatory network with memory loss. The threshold of X_1 is any number N. The threshold of all S_i is one.

Let the Boolean system associated with the network in Fig. S4 with $N = 2$ be:

$$\begin{aligned} S_1^+ &= C_1 && \\ S_i^+ &= S_{i-1} && \text{for } 1 < i \leq m \\ S_i^+ &= S_{i-1} \wedge (\neg X_1) && \text{for } m < i \leq k \\ X_1^+ &= \bigvee_{i,j \in K, i \neq j} (S_i \wedge S_j) && \end{aligned} \tag{1}$$

where $K = \{1, \cdots, k\}$.

We first prove in Lemma 2.2 - Lemma 2.5 that when $m - 2 \leq p \leq k - 1$, then all the trajectories pass through the state:

$$(S_1 \cdots S_k) = (\underbrace{10 \cdots 0}_{p}\underbrace{10 \cdots 0}_{k-p}) \text{ and } X_1 = 0. \tag{2}$$

For convenience, we call this state the *common initial state* of system (4).

Lemma 2.2. *Consider system (1), let the signal* $C_1 = (\overline{10 \cdots 0})$ *be periodic with period* p *and* $m \geq 7$*. If* $p = m - 2$*, then all the trajectories at steady-state pass through the common initial state.*

Proof. Note that the values of $(S_1, \cdots, S_m)$ are determined by the controller signal C_1 only.

Since $p = m - 2$, any given trajectory must pass a state where

$$(S_1 \cdots S_m) = (\underbrace{10 \cdots 0}_{p} 10).$$

Without loss of generality, we assume that this state occurs at Step 0. Because there are at least two "1"s in the sequence of S_i the **Rules** guarantee that, at Step 1:

$$(S_1 \cdots S_m) = (0 \underbrace{10 \cdots 0}_{p} 1) \text{ and } X_1 = 1.$$

At this step, there are still at least two "1"s in the sequence of S_i. Since an active X_1 inhibits all S_i for $i > m = p + 2$, it follows that at Step 2

$$(S_1 \cdots S_k) = (00 \underbrace{10 \cdots 0}_{p} \underbrace{00 \cdots}_{k-m}) \text{ and } X_1 = 1.$$

That is, there is only one "1" remaining in the sequence of S_i at this step. Which means, there are not enough active S_i to keep X_1 active in the next step. It follows that at Step 3,

$$(S_1 \cdots S_k) = (000 \underbrace{10 \cdots 0}_{p} \underbrace{00 \cdots}_{k-m-1}) \text{ and } X_1 = 0.$$

The number of "1"s will remain one until Step p, at which

$$(S_1 \cdots S_k) = (\underbrace{10 \cdots 0}_{p} \underbrace{10 \cdots 0}_{k-p}) \text{ and } X_1 = 0.$$

Then the lemma is proved. □

The same result holds when $p = m - 1$ as we prove next.

Lemma 2.3. *Consider system (1), let the signal* $C_1 = (\overline{10 \cdots 0})$ *be periodic with period* p *and* $m \geq 7$*. If* $p = m - 1$*, then all trajectories at steady-state goes through the common initial state.*

Proof. Since $m = p + 1$, similar to the proof for Lemma 2.2, we can assume that at Step 0,

$$(S_1 \cdots S_m) = (\underbrace{10 \cdots 0}_{p} 1).$$

There are at least two "1"s in the sequence of S_i regardless of the value of X_1 at this step. Since the threshold for activating X_1 is 2, at Step 1, $X_1 = 1$. Since in the next step an active X_1 inhibits all S_i for $i > m$, it follows that at Step 2

$$(S_1 \cdots S_k) = (00\underbrace{10\cdots 0}_{p}\underbrace{00\cdots}_{k-m-1}).$$

This means that only one of S_i has a value "1". The number of "1"s in the sequence of S_i remains one until Step p, at which

$$(S_1 \cdots S_k) = (\underbrace{10\cdots 0}_{p}\underbrace{10\cdots 0}_{k-p}) \text{ and } X_1 = 0.$$

Therefore, the lemma is proved. □

To prove Lemma 2.5, which gives a result similar to those in Lemmas 2.2 and 2.3 when $m \leq p \leq k-1$, we need the next Lemma.

Lemma 2.4. *In system (1), suppose the signal $C_1 = (\overline{10\cdots 0})$ is periodic with period p. If $p \leq k-1$, then X_1 cannot be silent, that is, $X_1 \neq (\overline{0})$ at steady state.*

Proof. We prove the lemma by contradiction. Suppose $X_1 = (\overline{0})$. Then S_i has no active inhibitor at any time step. It follows that the values of S_i is determined only by the periodic signal $C_1 = (\overline{10\cdots 0})$. This leads to

$$(S_1 \cdots S_k) = (\underbrace{10\cdots 0}_{p}\underbrace{10\cdots}_{k-p})$$

periodically. Since there are at least two "1"s in the sequence of S_i at such state, by the **Rules**, $X_1 = 1$ periodically, which is a contradiction. Hence, $X_1 \neq (\overline{0})$. □

Lemma 2.5. *In system (1), suppose the signal $C_1 = (\overline{10\cdots 0})$ is periodic with period p and $m \geq 7$. If $m \leq p \leq k-1$, then all the trajectories at steady-state pass through the common initial state.*

Proof. By Lemma 2.4, $X_1 \neq (\overline{0})$ at steady-state. For any given trajectory, there must exist a step at which $X_1 = 1$. Since $p \geq m$, there is at most one $S_i = 1$ for some $i \leq m$ and $S_i = 0$ for $m+1 \leq i \leq k$ at the next step. If $S_i = 0$ for all i, then after at most $p+1$ steps, the trajectory will reach the common initial state. Otherwise, if one of $S_i = 1$, then there will be a step for which $S_2 = 1$ and $S_i = 0$ for all $i \neq 2$. The number of "1"s in the sequence $\{S_i\}$ remains one until Step p, at which

$$(S_1 \cdots S_k) = (\underbrace{10\cdots0}_{p}\underbrace{10\cdots0}_{k-p}) \text{ and } X_1 = 0.$$

Hence, the lemma is proved.

□

Theorem 2.6. *In system (1), suppose the signal $C_1 = (\overline{10\cdots0})$ is periodic with period p and $m \geq 7$. Then*

(a) when $p \leq \frac{m}{2}$, $X_1 = (\overline{1})$ at steady state;

(b) when $\frac{m}{2} < p < m-2$, $X_1 = (\overline{\underbrace{1\cdots1}_{m-p}\underbrace{0\cdots0}_{2p-m}})$ at steady state;

(c) when $m-2 \leq p < k-1$, $X_1 = (\overline{11\underbrace{0\cdots0}_{p-2}})$ at steady state;

(d) when $p = k-1$, $X_1 = (\overline{\underbrace{10\cdots0}_{p}})$ at steady state;

(e) when $p \geq k$, X_1 is silent at steady state.

Proof. (a) When $p \leq \frac{m}{2}$, after at most m number of steps, at least two of S_i will have values of "1" at any following step. Because the threshold for activating X_1 is 2, by the **Rules**(a), $X_1 = (\overline{1})$ at steady state.

(b) When $\frac{m}{2} < p < m-2$, $(S_1 \cdots S_{p+1})$ is determined only by the control signal C_1. Without loss of generality, we can always assume that at Step 0,

$$(S_1 \cdots S_{p+1}) = (\underbrace{10\cdots0}_{p}1).$$

Then there are at least two "1"s in the sequence $(S_1 \cdots S_k)$, which will activate X_1 in the next step, i.e., at Step 1 $X_1 = 1$. Since $p+2 < m < 2p$, the values of S_i at Step 1 are

$$(0\underbrace{10\cdots0}_{p}\underbrace{10\cdots0}_{m-p-1}\underbrace{0\cdots0}_{k-m}).$$

The number of "1"s in the sequence $(S_1 \cdots S_k)$ remains exactly two from Step 2 to Step $m-p-1$, at which

$$(S_1 \cdots S_k) = (\underbrace{0\cdots0}_{m-p-1}\underbrace{10\cdots0}_{p}\underbrace{10\cdots0}_{k-m+1}) \text{ and } X_1 = 1.$$

It follows that at Step $m-p$,

$$(S_1 \cdots S_k) = (\underbrace{0\cdots0}_{m-p}\underbrace{10\cdots0}_{p}\underbrace{0\cdots0}_{k-m}) \text{ and } X_1 = 1$$

where there is only one "1" remaining in the sequence $(S_1 \cdots S_k)$. By the **Rules**, in the next step (Step $m-p+1$), $X_1 = 0$. This situation remains the same until the state returns to where it was at Step 0 and the pattern of states repeats. Hence, $X_1 = (\overline{\underbrace{1\cdots1}_{m-p}\underbrace{0\cdots0}_{2p-m}})$;

(c) When $m-2 \leq p < k-1$, by Lemmas 2.2 - 2.5, we can assume that at Step 0, the system is at the common initial state:

$$(S_1 \cdots S_k) = (\underbrace{10\cdots0}_{p}10\underbrace{0\cdots0}_{k-m}) \text{ and } X_1 = 0.$$

Then at Step 1,

$$(S_1 \cdots S_k) = (0\underbrace{10\cdots0}_{p}1\underbrace{0\cdots0}_{k-m}) \text{ and } X_1 = 1.$$

At Step 2,

$$(S_1 \cdots S_k) = (00\underbrace{10\cdots0}_{p}\underbrace{0\cdots0}_{k-m}) \text{ and } X_1 = 1.$$

By the **Rules** (a), $X_1 = 0$ at Step 3 since there is only one “1” in the sequence $(S_1 \cdots S_k)$. The number of “1”s in $(S_1 \cdots S_k)$ remains one until Step p at which

$$(S_1 \cdots S_k) = (\underbrace{10\cdots0}_{p}10\underbrace{0\cdots0}_{k-m}) \text{ and } X_1 = 0$$

which is the same as the values at Step 0. Hence, at steady state $X_1 = (\overline{11\underbrace{0\cdots0}_{p-2}})$.

(d) When $p = k-1$, by Lemma 2.5, we can assume that at Step 0, the system is at the common initial state:

$$(S_1 \cdots S_k) = (\underbrace{10\cdots0}_{p}1) \text{ and } X_1 = 0.$$

Then at Step 1, the state values become

$$(S_1 \cdots S_k) = (0\underbrace{10\cdots0}_{p}) \text{ and } X_1 = 1.$$

It follows that at Step 2, $X_1 = 0$ and the number of “1”s in the sequence $(S_1 \cdots S_k)$ remains one until Step p, at which the state returns to where it was at Step 0. Hence, at steady state $X_1 = (\overline{\underbrace{10\cdots0}_{p}})$.

(e) When $p \geq k$, after at most k steps, there is no more than one “1” in the sequence of $(S_1 \cdots S_k)$ at any time step. Thus, by the **Rules**, $X_1 = (\overline{0})$.

□

The results in Theorem 2.6 are illustrated in Fig. S5.

A similar result holds when the threshold $N > 2$, as we prove next.

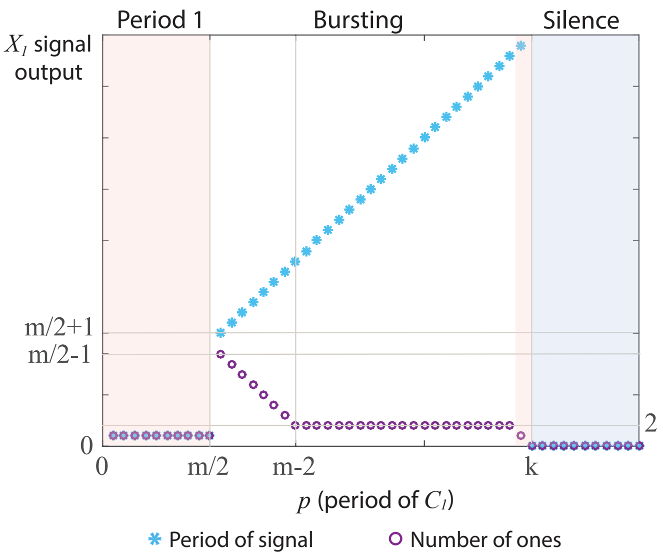

Figure S5: The output of the excitatory network with memory loss (X_1, see Fig. S4) as a function of p (the period of C_1) when the threshold of X_1 is two and $m \geq 7$ (see Theorem 2.6). X_1 is periodic with period 1 when $p \leq m/2$; bursting with period p and $m-p$ consecutive "1"s when $\frac{m}{2} < p < m-2$; bursting with period p and 2 consecutive "1"s when $m-2 \leq p < k-1$; periodic with period p when $p = k-1$ and silent when $p \geq k$.

Let the Boolean system associated with the network in Fig. S4 be:

$$\begin{aligned} S_1^+ &= C_1 && \\ S_i^+ &= S_{i-1} && \text{for } 1 < i \leq m \\ S_i^+ &= S_{i-1} \wedge (\neg X_1) && \text{for } m < i \leq k \\ X_1^+ &= \bigvee_{i_j \in K} (S_{i_1} \wedge S_{i_2} \wedge \cdots \wedge S_{i_N}) && \end{aligned} \tag{3}$$

where $K = \{1, \cdots, k\}$ and $N \geq 2$.

We call the state at which

$$(S_1 \cdots S_k) = (\underbrace{\overbrace{10 \cdots 0}^{N-1}}_{p}\underbrace{10 \cdots 0}_{k-p}) \text{ and } X_1 = 0$$

the *common initial state* of (3).

Lemma 2.7. *If $p = \frac{m-1}{N-1}$ or $\frac{m-2}{N-1}$ and $m \geq 7$, then all the trajectories of System (3) pass through its common initial state.*

Proof. Since $m = (N-1)p + 1$ or $m = (N-1)p + 2$, $(S_1 \cdots S_{(N-1)p+1})$ is determined only by the periodic control signal C_i. Then there must be a step at which

$$(S_1 \cdots S_{(N-1)p+2}) = (\overbrace{\underbrace{10 \cdots 0}_{p}}^{N-1} 10).$$

For convenience, we call this step, Step 0. Note that the number of "1"s in the sequence $(S_1 \cdots S_k)$ is at least N. Since the threshold for activating X_1 is N, $X_1 = 1$ at Step 1. Because X_1 inhibits all S_i with $i > m$,

$$(S_1 \cdots S_k) = (00 \overbrace{\underbrace{10 \cdots 0}_{p}}^{N-1} \underbrace{0 \cdots 0}_{k-m-1})$$

at Step 2, if $m = (N-1)p + 1$ or,

$$(S_1 \cdots S_k) = (000 \overbrace{\underbrace{10 \cdots 0}_{p}}^{N-1} \underbrace{0 \cdots 0}_{k-m-1})$$

at Step 3, if $m = (N-1)p + 2$. In both these cases there are only $N-1$ number of "1"s in the sequence $(S_1 \cdots S_k)$. It follows that $X_1 = 0$ at Step 3 if $m = (N-1)p + 1$ or at Step 4 if $m = (N-1)p + 2$. The number of "1"s in the sequence $(S_1 \cdots S_k)$ remains $N-1$ until Step p, at which

$$(S_1 \cdots S_k) = (\overbrace{\underbrace{10 \cdots 0}_{p}}^{N-1} 1 \underbrace{0 \cdots 0}_{k-m}) \text{ and } X_1 = 0$$

if $m = (N-1)p + 1$ or,

$$(S_1 \cdots S_k) = (\overbrace{\underbrace{10 \cdots 0}_{p}}^{N-1} 10 \underbrace{0 \cdots 0}_{k-m}) \text{ and } X_1 = 0$$

if $m = (N-1)p + 2$. In both cases, all the trajectories pass through the common initial state of the system. □

Lemma 2.8. *In system (3), if $p \leq \frac{k-1}{N-1}$, then $X_1 \neq (\overline{0})$.*

Proof. We prove the lemma by contradiction. We assume that $X_1 = (\overline{0})$ under the given condition $p \leq \frac{k-1}{N-1}$, i.e. $k \geq (N-1)p + 1$. Then for every number of steps, there will always be a recent step (i.e. within the previous k steps) for which

$$(S_1 \cdots S_k) = (\overbrace{\underbrace{10\cdots0}_{p}}^{N-1} 1 \quad \underbrace{0\cdots0}_{k-(N-1)p-1} \quad)$$

where there are N number of "1"s in the sequence $(S_1 \cdots S_k)$. This implies that $X_1 = 1$ periodically, which is a contradiction. □

Lemma 2.9. *For system (3), if $\frac{m}{N-1} \leq p \leq \frac{k-1}{N-1}$ and $m \geq 7$, then all trajectories go through the common initial state of the system.*

Proof. Note that $\frac{m}{N-1} \leq p \leq \frac{k-1}{N-1}$ means $m \leq (N-1)p \leq k-1$. By Lemma 2.9, $X_1 \neq (\overline{0})$. It follows that for any given trajectory, there must exist a step at which $X_1 = 1$. Without loss of generality, we assume that occurs at Step 0. Since X_1 inhibits all S_i for $i > m$, there are at most $N-1$ number of $S_i = 1$ for $1 \leq i \leq m$ at Step 1. It follows that $X_1 = 0$ at Step 2. The number of S_i having a value of "1" remains $\leq N-1$ for some steps after until the trajectory reaches the common initial state of the system. Then the lemma is proved. □

Theorem 2.10. *In system (3), let the signal $C_1 = (\overline{10\cdots0})$ be periodic with period p and $m \geq 7$. Then*

(a) when $p \leq \frac{m}{N}$, $X_1 = (\overline{1})$ at steady state;

(b) when $\frac{m}{N} < p < \frac{m-2}{N-1}$, $X_1 = (\overline{\underbrace{1\cdots1}_{s}\underbrace{0\cdots0}_{p-s}})$ at steady state , where $s = m-(N-1)p$;

(c) when $\frac{m-2}{N-1} \leq p < \frac{k-1}{N-1}$, $X_1 = (\overline{11\underbrace{0\cdots0}_{bp-2}})$ at steady state, where $b = N - \lceil\frac{m-2}{p}\rceil$.

(d) when $p = \frac{k-1}{N-1}$, $X_1 = (\underbrace{\overline{10\cdots0}}_{p})$ *at steady state;*

(e) when $p > \frac{k-1}{N-1}$, X_1 *is silent at steady state.*

Proof. (a) When $p \leq \frac{m}{N}$, i.e., $Np \leq m$, after a sufficient number of initial steps, there always exists at least N numbers of S_i having a value of "1". By the **Rules** (a), $X_1 = (\overline{1})$ at steady state.

(b) When $\frac{m}{N} < p < \frac{m-2}{N-1}$, (i.e. $(N-1)p + 2 < m < Np$), $(S_1 \cdots S_m)$ is controlled only by C_1. So there must exist a step at which

$$(S_1 \cdots S_m) = (\overbrace{\underbrace{10\cdots0}_{p}\cdots}^{N-1}\underbrace{10\cdots0}_{s})$$

where $s = m - (N-1)p$. There are N number of "1"s in the sequence $(S_1 \cdots S_k)$. Without loss of generality, we assume it is Step 0. By the **Rules**, at Step 1

$$(S_1 \cdots S_m) = (0\overbrace{\underbrace{10\cdots0}_{p}\cdots}^{N-1}\underbrace{10\cdots0}_{s-1}) \text{ and } X_1 = 1.$$

Then until Step $s = m - (N-1)p$, there are exactly N number of S_i having a value of "1". At Step $s-1$,

$$(S_1 \cdots S_m) = (\underbrace{0\cdots0}_{s-1}\overbrace{\underbrace{10\cdots0}_{p}\cdots}^{N-1}1) \text{ and } X_1 = 1$$

At Step s,

$$(S_1 \cdots S_k) = (\underbrace{0\cdots0}_{s}\overbrace{\underbrace{10\cdots0}_{p}\cdots}^{N-1}0\cdots0)$$

because of the inhibition of X_1 acting on all S_i for $i > m$. This results in a state with only $N-1$ number of "1" in the sequence of $(S_1 \cdots S_k)$. The number of "1"s remains $N-1$ for the following $p - s = Np - m$ steps. At Step p the state goes back to the state at Step 0. This pattern of activity repeats every p steps. By the **Rules**, $X_1 = (\overline{\underbrace{1\cdots1}_{s}\underbrace{0\cdots0}_{p-s}})$.

(c) When $\frac{m-2}{N-1} \le p < \frac{k-1}{N-1}$ (i.e. $m-2 \le (N-1)p < k-1$), by Lemmas 2.7 and 2.9, we can assume at Step 0

$$(S_1 \cdots S_k) = (\overbrace{\underbrace{10\cdots0}_{p}}^{N-1}10\cdots0) \text{ and } X_1 = 0$$

Then at Step 1,

$$(S_1 \cdots S_k) = (0\overbrace{\underbrace{10\cdots0}_{p}}^{N-1}10\cdots0) \text{ and } X_1 = 1$$

Let $r = \lceil \frac{m-2}{p} \rceil \le N-1$. Since X_1 inhibits all S_i for $i > m$,

$$(S_1 \cdots S_k) = (00\overbrace{\underbrace{10\cdots0}_{p}}^{r-1}10\cdots0)$$

at Step 2. Note that there are $r(< N)$ number of "1"s in the sequence. It follows that $X_1 = 0$ at Step 3 and remains "0" for the next $(N-r)p-2$ steps. The state values go back to the common initial state at Step $(N-r)p$ and the patterns of the states just described repeat. Hence, at steady state $X_1 = (\overline{11\underbrace{0\cdots0}_{bp-2}})$, where $b = N-r$.

(d) The proof is similar to the proof of Theorem 2.6 d). More specifically, by Lemma 2.9 we can assume that the system is in its *common initial state*. That is,

$$(S_1 \cdots S_k) = (\overbrace{\underbrace{10\cdots0}_{p}}^{N-1}1) \text{ and } X_1 = 0$$

Then, at Step 1

$$(S_1 \cdots S_k) = (0\overbrace{\underbrace{10\cdots0}_{p}}^{N-1}) \text{ and } X_1 = 1$$

At Step 2 $X_1 = 0$ and the number of signals S_i with value "1" in $(S_1 \cdots S_k)$ remains $N-1$ till Step p. At Step $p+1$ the state values of the sequence returns to the pattern at Step "0". Thus, the steady state $X_1 = (\overline{\underbrace{10\cdots0}_{p}})$.

(e) The proof is similar to the one given for Theorem 2.6 e): since $p > \frac{k-1}{N-1}$, at any step after at most k steps, the sequence $(S_1 \cdots S_k)$ has no more than $N-1$ number signals S_i with value "1". Consequently, by **Rules** $X_1 = (\overline{1})$.

□

Theorem 2.10 can be illustrated by the diagram in Fig. S6.

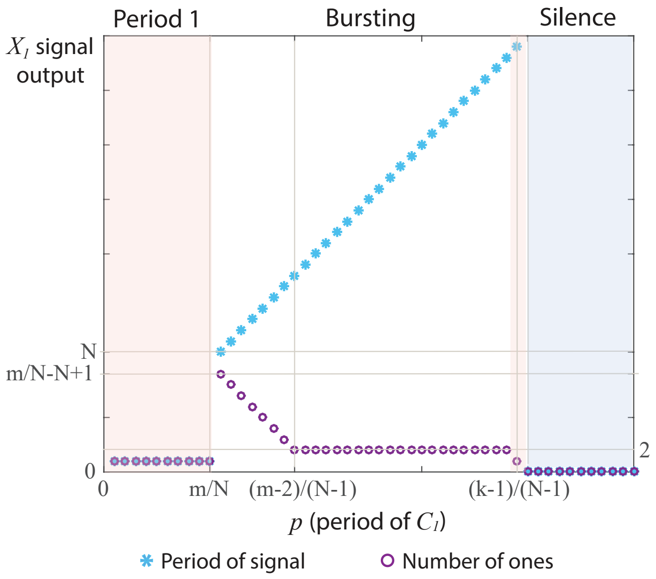

Figure S6: The output of the excitatory network with memory loss (X_1, see Fig. S4) as a function of p (the period of C_1) when the threshold of X_1 is N and $m \geq 7$ (see Theorem 2.10). X_1 is periodic with period 1 when $p \leq m/N$, bursting with period p and $m-(N-1)p$ consecutive ones when $\frac{m}{N} < p < \frac{m-2}{N-1}$, bursting with period p and 2 consecutive ones when $\frac{m-2}{N-1} \leq p < \frac{k-1}{N-1}$, periodic with period p when $p = \frac{k-1}{N-1}$ and silent when $p > \frac{k-1}{N-1}$.

As can be seen when comparing Figs. S5, S6 and Fig. 1, Panel A (main article), erasing some of the memory leads to an increase in the bursting region. However, for a large region of p values, the bursting is limited to two consecutive "1"s within a burst (this number can increase if the threshold for excitation of X_1 increases but this will then reduce the bursting region as seen in Theorem 2.10). Furthermore, if $m < 4$ the period of bursting becomes longer ($2p$ in

most of the cases), and if $m \leq 2$, the periodic trajectory with period 1 does not persist. The fact that a large portion of the bursting region consists of bursts with only two consecutive "1"s is limiting - it gives a very short inspiration time. We therefore look at another modification of this network.

2.3 Excitatory network with memory loss and self excitation ($N > 2$)

In this section, we consider the network in Sec. 2.2, but add a self-excitation loop to node X_1 as shown in Fig. S7. The case $N = 2$ is discussed in Methods, main article. Here we consider the situation where the threshold for activating X_1 is $N > 2$. More specifically, let the Boolean system associated with the network in Fig. S7 be:

$$\begin{aligned}
S_1^+ &= C_1 && \\
S_i^+ &= S_{i-1} && \text{for } 1 < i \leq m \\
S_i^+ &= S_{i-1} \wedge (\neg X_1) && \text{for } m < i \leq k \\
X_1^+ &= \bigvee_{i_j \in K} ((S_{i_1} \wedge S_{i_2} \cdots \wedge S_{i_N}) \vee (S_{i_1} \wedge S_{i_2} \cdots \wedge S_{i_{N-1}} \wedge I_1)) && \\
I_1^+ &= X_1 &&
\end{aligned} \tag{4}$$

where $K = \{1, \cdots, k\}$.

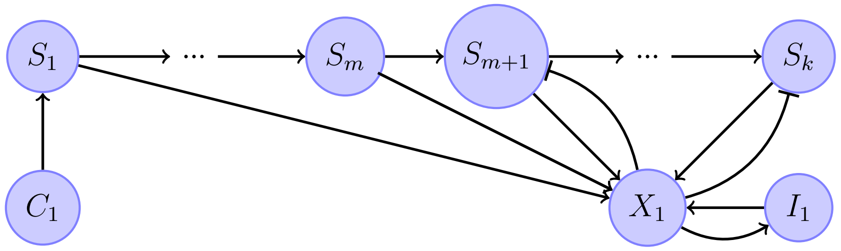

Figure S7: Excitatory network with memory loss and self excitation loop. The threshold of X_1 is any number N. The threshold of all S_i is one.

Lemma 2.11. *In system (4), when $p \leq \frac{k-1}{N-1}$ and $N \geq 2$, $X_1 \neq (\bar{0})$ at steady state.*

Proof. We prove the lemma by contradiction. Suppose $X_1 = (\bar{0})$ at steady state. Then

$(S_1 \cdots S_k)$ are determined only by the periodic control signal C_1. It follows that

$$(S_1 \cdots S_k) = (\overbrace{\underbrace{10\cdots 0}_{p}}^{N-1} 10\cdots 0)$$

periodically, where there are at least N number of "1"s in the sequence. This implies that $X_1 = 1$ periodically. This is a contradiction. □

Lemma 2.12. *In system (4), when $p \geq \frac{m+1}{N-1}$ and $N \geq 2$, $X_1 \neq (\overline{1})$ at steady state.*

Proof. We prove the lemma by contradiction. Suppose $X_1 = (\overline{1})$, then $I_1 = (\overline{1})$ and $S_i = 0$ for all $i > m$. On one hand, in order to have $X_1 = (\overline{1})$, there must be at least $N - 1$ number of S_i having a value of "1" since the threshold for activating X_1 is N and $I_1 = 1$. On the other hand, because $(N-1)p \geq m+1$, which is equivalent to $p \geq m - (N-2)p + 1$, the values of S_i nodes will be periodically equal to

$$(S_1 \cdots S_k) = (\underbrace{0\cdots 0}_{s} \overbrace{\underbrace{10\cdots 0}_{p}}^{N-2} 0\cdots 0)$$

where there are **exactly** $N-2$ number of "1"s in the sequence $(S_1 \cdots S_k)$ and $s = m-(N-2)p$ (i.e., $s + (N-2)p = m$). This means $X_1 = 0$ periodically, which is a contradiction. So $X_1 \neq (\overline{1})$. □

Lemma 2.13. *In system (4), when $N \geq 3$ and $p \geq \frac{m}{N-2}$, i.e. $(N-2)p \geq m$, there exist two consecutive "0"s in the trajectory of X_1 at steady state.*

Proof. Suppose there are no consecutive "0"s in the trajectory of X_1 at steady state. Then the pattern "101" must occur in the trajectory of X_1. Furthermore, $S_i = 0$ for any $i > m+1$ since $X_1 = 1$ for at least every two steps and it inhibits all S_i for $i > m$. Without loss of generality, we assume that at Steps 1, 2 and 3, the value of $X_1 = 1, 0,$ and 1 respectively. Since $(N-2)p \geq m$ and X_1 inhibits all S_i for $i > m$, there are at most $N-2$ number of S_i having a value of "1"

at Step 2. In addition, $I_1 = 1$ at Step 2. Thus, the number of activators of X_1 with value "1" is $N-1$. Because the threshold to activate X_1 is N, $X_1 = 0$ at Step 3. This contradicts the assumption that $X_1 = 1$ at Step 3. Hence, the trajectory of X_1 contains consecutive "0"s. $\square$

Lemma 2.14. *In system (4), when $N \geq 3$ and $\frac{m+2}{N-1} \leq p < \frac{m}{N-2}$, i.e. $(N-1)p \geq m+2$ and $(N-2)p < m$, there exist two consecutive "0"s in the trajectory of X_1 at steady state.*

Proof. Suppose there are no consecutive zeros in the trajectory of X_1 at steady state. Since by Lemma 2.12 $X_1 \neq (\overline{1})$, the pattern 101 must occur in the trajectory of X_1. It follows that $S_i = 0$ for any $i > m+1$ since $X_1 = 1$ for at least every two steps and it inhibits all S_i for $i > m$. Without loss of generality, we assume that at Steps 1, 2 and 3, the value of $X_1 = 1, 0,$ and 1 respectively. Because of the inhibition from X_1 to S_i for $i > m$ and because $(N-1)p \geq m+2$, at Step 2, the number of S_i having the value of "1" is at most $N-1$. Suppose there are at most $N-2$ number of S_i having a value of "1" at Step 2. Then $X_1 = 0$ at Step 3, which is a contradiction. Hence, there are exactly $N-1$ number of S_i having a value of "1". Without loss of generality, we assume that at Step 2

$$(S_1 \cdots S_m S_{m+1}) = (0\,\overbrace{\underbrace{10\cdots0}_{p}}^{N-2}\,10\cdots00)$$

where there are exactly N-1 number of "1"s in the sequence $(S_1 \cdots S_k)$. Note that $p \geq m - (N-2)p + 2$. Let $s = m - (N-2)p$, then $p \geq s+2$. It follows that up to Step s at which

$$(S_1 \cdots S_m S_{m+1}) = (\underbrace{0\cdots0}_{s-1}\,\overbrace{10\cdots0}^{N-2}\,10)$$

where there are exactly $N-1$ number of S_i having a value of "1" and the values of X_1 and I_1 alternate between 0 and 1. Hence, the values of (X_1, I_1) at this step can be either $(1,0)$ or $(0,1)$. We will show next that both cases lead to consecutive "0"s in the trajectory of X_1.

Case I: $(X_1, I_1) = (1, 0)$ at Step s. Then at Step $s+1$,

$$(S_1 \cdots S_m S_{m+1}) = (\underbrace{0 \cdots 0}_{s} \overbrace{10 \cdots 0}^{N-2} 0) \text{ and } (X_1, I_1) = (0, 1)$$

where there are exactly $N-2$ number of S_i with a value "1". This is because $X_1 = 1$ in the previous step and $p \geq m+2-(N-2)p = s+2$. It follows that $X_1 = 0$ at Step $s+2$, which contradicts with the assumption that there are no consecutive zeros in the trajectory of X_1.

Case II: $(X_1, I_1) = (0, 1)$ at Step s. At Step $s+1$,

$$(S_1 \cdots S_m S_{m+1}) = (\underbrace{0 \cdots 0}_{s} \overbrace{10 \cdots 0}^{N-2} 1) \text{ and } (X_1, I_1) = (1, 0)$$

where there are exactly $N-1$ number of "1"s in the sequence $(S_1 \cdots S_k)$. By the **Rules,** at Step $s+2$

$$(S_1 \cdots S_m S_{m+1}) = (\underbrace{0 \cdots 0}_{s+1} \overbrace{10 \cdots 0}^{N-2}) \text{ and } (X_1, I_1) = (0, 1)$$

where there are exactly $N-2$ number of "1"s in the sequence $(S_1 \cdots S_k)$. Then at Step $s+3$, $X_1 = 0$ since X_1 has only $N-1$ number of activators having a value of "1" ($N-2$ number of $S_i = 1$ and $I_1 = 1$). Again, two consecutive "0"s occur in the trajectory of X_1. This is a contradiction.

In summary, the trajectory of X_1 has two consecutive "0"s at steady state. □

Lemma 2.15. *In system (4), suppose there are two consecutive "0"s in the trajectory of X_1 at steady state. Then all trajectories pass through*

$$(S_1 \cdots S_k) = (\underbrace{\overbrace{10 \cdots 0}^{N-1}}_{p} 10 \cdots 0) \textit{ and } (X_1, I_1) = (0, 0)$$

Proof. When the trajectory of X_1 has two consecutive zeros at steady state, the first state that enables to reactivate X_1 after these two consecutive steps must be

$$(S_1 \cdots S_k) = (\underbrace{\overbrace{10 \cdots 0}^{N-1}}_{p} 10 \cdots 0) \text{ and } (X_1, I_1) = (0, 0)$$

The lemma is proved. □

Theorem 2.16. *In system (4), suppose $N > 2$,*

(a) when $p < \frac{m}{N-1}$, $X_1 = (\overline{1})$ at steady state;

(b) when $\frac{m+2}{N-1} \leq p < \frac{m-2}{N-2}$ and $p \leq \frac{k-2}{N-1}$, $X_1 = (\overline{\underbrace{1\cdots1}_{s}\underbrace{0\cdots0}_{2p-s}})$ where $s = m - (N-2)p$;

(c) when $\max\{4, \frac{m-1}{N-2}\} \leq p \leq \frac{k-2}{N-1}$, $X_1 = (\overline{11\underbrace{0\cdots0}_{bp-2}})$ with $b = N - \lceil \frac{m-2}{p} \rceil$.

(d) if $p \geq \frac{k}{N-2}$, then X_1 is silent at steady state.

Proof. (a) When $p < \frac{m}{N-1}$, we have $(N-1)p < m$. It follows that at steady state, there are at least $N-1$ number of S_i with a value of "1" at any time step. In addition, suppose $X_1 = 1$ for two consecutive steps, say at Steps 1 and 2. Then $I_1 = 1$ at Step 2. By the **Rules**, $(X_1, I_1) = (1, 1)$ at Step 3 and any step afterwards. Hence, $X_1 = (\overline{1})$.

Next, we show that at steady state, there must exist two consecutive steps at which $X_1 = 1$.

Since $(N-1)p < m$, at steady state the activity of S_i for $i \leq m$ is determined uniquely by C_1. So there always exists a step at which

$$(S_1 \cdots S_{(N-1)p+1}) = (\overbrace{\underbrace{\overline{10\cdots0}}_{p}}^{N-1}1)$$

Without loss of generality, we assume that this occurs at Step 0. Note that there are at least N number of "1"s in the sequence $(S_1 \cdots S_k)$ and the values of (X_1, I_1) can be either $(1, 0)$, $(0, 1)$ or $(0, 0)$.

We show next that each of these cases leads to two consecutive "1"s in the trajectory of X_1.

Case I. Suppose $(X_1, I_1) = (1, 0)$. Then at Step 1,

$$(S_1 \cdots S_{(N-1)p+1}) = (0\overbrace{\underbrace{10\cdots 0}_{p}}^{N-1}),\ (X_1, I_1) = (1, 1)$$

where there are at least N-1 number of "1"s in the sequence $(S_1 \cdots S_k)$. Hence, we found two consecutive "1"s in the trajectory of X_1.

Case II. Suppose $(X_1, I_1) = (0, 1)$. Then at Step 1,

$$(S_1 \cdots S_{(N-1)p+1}) = (0\overbrace{\underbrace{10\cdots 0}_{p}}^{N-1}1\cdots), (X_1, I_1) = (1, 0)$$

where there are at least N number of "1"s in the sequence $(S_1 \cdots S_k)$. It follows that at Step 3, $X_1 = 1$. So again, there are two consecutive "1"s in the trajectory of X_1.

Case III. Finally, suppose $(X_1, I_1) = (0, 0)$, we can easily see that at Step 1 and 2, $X_1 = 1$. Therefore, each of these three cases leads to two consecutive "1"s in the trajectory of X_1. Thus, the trajectory of $X_1 = (\overline{1})$ at steady state.

(b) If $\frac{m+2}{N-1} \leq p < \frac{m-2}{N-2}$, then $(N-2)p \leq m-2$ and $(N-1)p \geq m+2$, which is equivalent to $p \geq m + 2 - (N-2)p = s + 2$. By Lemmas 2.14 and 2.13, the trajectory of X_1 contains two consecutive "0"s. Then by Lemma 2.15, without loss of generality we can assume that at Step 0,

$$(S_1 \cdots S_k) = (\overbrace{\underbrace{10\cdots 0}_{p}}^{N-1}1\cdots 0) \text{ and } (X_1, I_1) = (0, 0) \tag{5}$$

At Step 1,

$$(S_1 \cdots S_k) = (0\overbrace{\underbrace{10\cdots 0}_{p}}^{N-1}1\cdots 0) \text{ and } (X_1, I_1) = (1, 0) \tag{6}$$

Note the states at (5) and (6) require the condition $p \leq \frac{k-2}{N-1}$, i.e. $p(N-1)+2 \leq k$. At Step 2, $(X_1, I_1) = (1,1)$ and remains unchanged up to Step $s-1$. At Step $s-1$,

$$(S_1 \cdots S_m) = (\underbrace{0 \cdots 0}_{s-1}\overbrace{10 \cdots 0}^{N-2}1), (X_1, I_1) = (1,1)$$

where there are exactly $N-1$ number of "1"s in the sequence $(S_1 \cdots S_k)$. From Step s to Step $p-1 \geq s+1$, the number of S_i with a value of "1" is $N-2$, which effectively leaves $X_1 = 0$ for at least two consecutive steps. Then the state goes back to the state of Step 1 at Step $2p$. This pattern of activity repeats every $2p$ steps. Hence, $X_1 = (\overline{\underbrace{1 \cdots 1}_{s}\underbrace{0 \cdots 0}_{2p-s}})$.

(c) By $p \geq \frac{m-1}{N-2}$, we have $(N-2)p \geq m-1$. Since $p \geq 4$, $(N-2)p+p \geq (m-1)+4 > m+2$. Therefore, $(N-1)p \geq m+2$. By Lemmas 2.13 - 2.15, we can assume at Step 0,

$$(S_1 \cdots S_k) = (\overbrace{\underbrace{10 \cdots 0}_{p}10 \cdots 0}^{N-1}) \text{ and } (X_1, I_1) = (0,0)$$

Then at Step 1,

$$(S_1 \cdots S_k) = (0\overbrace{\underbrace{10 \cdots 0}_{p}10 \cdots 0}^{N-1}) \text{ and } (X_1, I_1) = (1,0)$$

At Step 2,

$$(S_1 \cdots S_k) = (00\overbrace{\underbrace{10 \cdots 0}_{p}10 \cdots 0}^{\lceil \frac{m-2}{p} \rceil - 1}), (X_1, I_1) = (1,1)$$

where there are exactly $\lceil \frac{m-2}{p} \rceil$ number of S_i with a value of "1". Note $\frac{m-2}{p} < \frac{m-1}{p} \leq N-2$ since $(N-2)p \geq m-1 > m-2$. It follows that in the next $p-3 \geq 1$ step(s), X_1 has at most $N-1$ (including the activator I_1) number of activators having a value of "1". More specifically, at Step 4, $(X_1, I_1) = (0,0)$. The first state after Step 4 that enables activating X_1 must be the same as the state at Step 0 which takes place at Step $(N - \lceil \frac{m-2}{p} \rceil)p$. Hence, $X_1 = (\overline{11\underbrace{0 \cdots 0}_{bp-2}})$ where $b = N - \lceil \frac{m-2}{p} \rceil$.

(d) When $p \geq \frac{k}{N-2}$, $(N-2)p \geq k$. So at steady state, the number of S_i having a value of "1" is at most $N-2$. This means X_1 has at most $N-1$ number of activators having a value of "1". By the **Rules** (a), $X_1 = (\overline{0})$.

□

From Theorem 2.16, we can see that for most of the combinations of m and k, there exists a unique steady state. Fig. S8 illustrates Theorem 2.16 with initial condition

$$(S_1 \cdots S_k) = (\underbrace{\overbrace{10\cdots0}^{N-1}}_{p} 10\cdots0),\ X_1 = 0 \text{ and } I_1 = 0$$

and $N = 5$.

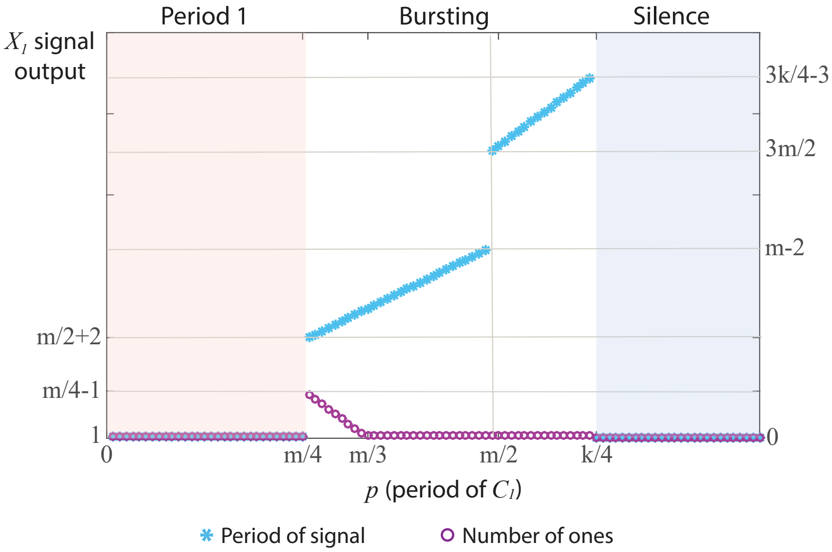

Figure S8: The output of the excitatory network with memory loss and self excitation (X_1, see Fig. S7) as a function of p (the period of C_1) when the threshold of X_1 is $N = 5$ (see Theorem 2.16). X_1 is periodic with period 1 when $p < \frac{m}{4}$, bursting with period $2p$ and $m-3p$ consecutive "1"s when $\frac{m}{4} \leq p < \frac{m}{3}$, bursting with period $2p$ and two consecutive "1"s when $\frac{m}{3} < p < \frac{m}{2}$; bursting with with period $3p$ and two consecutive "1"s when $\frac{m}{2} < p < \frac{k}{4}$; and silent when $p < \frac{k}{4}$.

There are two striking differences between Fig. 1, Panel D and Fig. S8. The first is that the number of consecutive "1"s within the bursting is m in Fig. 1, Panel D, for all values of p for which bursting occurs. In contrast, in Fig. S8, when the period of the control signal C_1 changes from $m/4$ to $m/3$ the number of consecutive "1"s within a burst varies from $m/4 - 1$ to 2 and remains 2 until the end of the bursting region. The second is that the period of bursting in Fig. S8 increases suddenly when $p = m/2$.

3 Structure of the larger network

In this section we provide more information about the structure of the network shown in Fig. 2 of the main article.

The network around X_1 is shown in Fig. S9. The inhibition from X_3 and X_4 is achieved by suppressing S_i^1 for all i. As a consequence of the application of the **Rules** when either X_3 or X_4 is active, the activity of X_1 will be turned off in the following step.

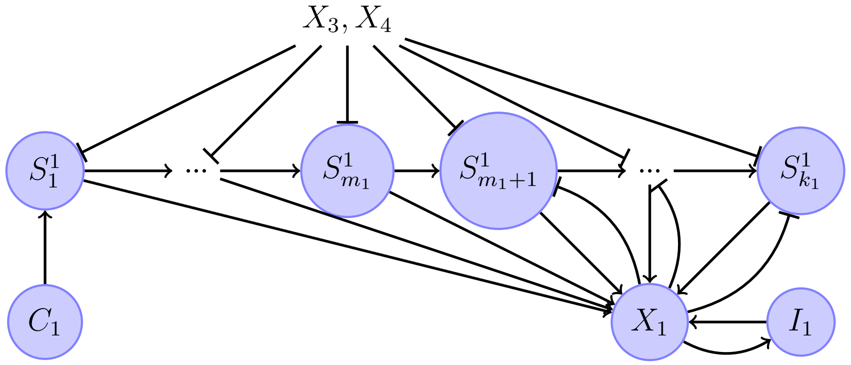

Figure S9: The network for X_1 (see Fig. 2 in the main article). The threshold for activating X_1 is 2. To simulate the 3-phase pattern, the period of C_1 is chosen such that X_1 is in spiking mode when it is isolated. Specifically, p_1 (period of C_1)$< m_1$. X_1 is turned off by X_4 or X_3 which inhibit all the memory nodes of X_1.

The network around X_3 in Fig. 2 of the main article, is almost the same as that of X_1 except that X_3 is not influenced by other nodes as shown in Fig. S10. This network is the same as the

one shown in Fig.1 (B) in the main text. Different periodic signals from C_3 lead to different rhythmicity in X_3 as shown in Fig.1 (D). This means that X_3 is quiet when $p_3 \geq k_3$, bursting when $m_3 < p_3 < k_3$ and spiking when $p_3 \leq m_3$, where p_3 is the period of C_3.

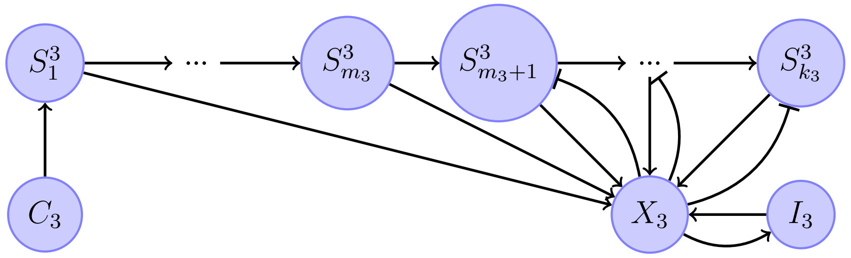

Figure S10: The network for X_3 (see Fig. 2 in the main article). The threshold for activating X_3 is 2. To simulate the 3-phase pattern, we choose the period of C_3 such that X_3 is in bursting mode when it is in isolation. Specifically, $m_3 < p_3 < k_3$ where p_3 is the period of C_3.

The network around X_4 is shown in Fig. S11. It takes the form of Fig.1 (A) with inhibitory signals from X_3 and X_4 that suppress all of S_i^4.

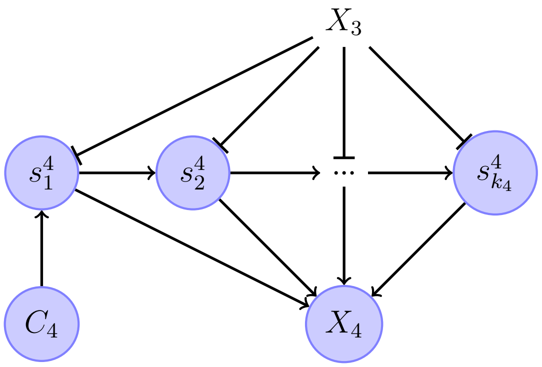

Figure S11: The network for X_4 (see Fig. 2 in the main article). The threshold for activating X_4 is 3 (the reason for making the threshold to activate X_4 higher than the threshold to activate X_1 is explained in the Section: Mechanisms of pattern generation, main article. To simulate the 3-phase pattern, we choose the period of C_4 such that X_4 is in spiking mode when it is isolated (i.e. $p_4 < \frac{k_4}{3}$, see Fig. 1 Panel C). X_4 receives inhibitory signals from X_3 by shutting off all the memory nodes of X_4.
